# An assessment of climate change vulnerability for Important Bird Areas in the Bering Sea and Aleutian Arc

**DOI:** 10.1371/journal.pone.0214573

**Published:** 2019-04-17

**Authors:** Melanie A. Smith, Benjamin K. Sullender, William C. Koeppen, Kathy J. Kuletz, Heather M. Renner, Aaron J. Poe

**Affiliations:** 1 Audubon Alaska, Anchorage, Alaska, United States of America; 2 Axiom Data Science, Anchorage, Alaska, United States of America; 3 US Fish and Wildlife Service, Anchorage, Alaska, United States of America; 4 Alaska Maritime National Wildlife Refuge, US Fish and Wildlife Service, Homer, Alaska, United States of America; Hawaii Pacific University, UNITED STATES

## Abstract

Recently available downscaled ocean climate models for the Bering Sea and Aleutian Arc offer the opportunity to assess climate vulnerability for upper trophic level consumers such as marine birds. We analyzed seasonal and annual spatial projections from three climate models for two physical climate variables (seawater temperature and sea ice) and three forage variables (large copepods, euphausiids, and benthic infauna), comparing projected conditions from a recent time period (2003–2012) to a future time period (2030–2039). We focused the analyses on core areas within globally significant Important Bird Areas, and developed indices of the magnitude of projected change and vulnerability agreement among models. All three climate models indicated a high degree of change for seawater temperature warming (highest in the central and eastern Aleutian Islands) and ice loss (most significant in the eastern Bering Sea) across scales, and we found those changes to be significant for every species and virtually every core area assessed. There was low model agreement for the forage variables; while the majority of core areas were identified as climate vulnerable by one or more models (72% for large copepods, 73% for euphausiids, and 94% for benthic infauna), very few were agreed upon by all three models (only 6% of euphausiid-forager core areas). Based on the magnitude-agreement score, euphausiid biomass decline affected core areas for fulmars, gulls, and auklets, especially along the outer shelf and Aleutian Islands. Benthic biomass decline affected eiders along the inner shelf, and large copepod decline was significant for storm-petrels and auklets in the western Aleutians. Overall, 12% of core areas indicated climate vulnerability for all variables assessed. Modeling and interpreting biological parameters to project future dynamics remains complex; the strong signal for projected physical changes raised concerns about lagged responses such as distribution shifts, breeding failures, mortality events, and population declines.

## Introduction

Climate change has major ecological effects on both terrestrial and marine systems [1]. Globally, the measurable impacts of climate change on oceans include decreased productivity, altered food web dynamics, and shifts in species’ distributions [2, 3]; these changes are often attributed to long-term increases in ocean temperatures (e.g., [4, 5]). Changes in temperature may also have cascading effects on seabirds by indirect mechanisms that alter water column characteristics [6] and food webs, resulting in changes in prey availability [7–9]. Effects on food webs may be further confounded by other local environmental drivers, such as altering currents or upwelling regimes [10], effects from commercial fishing activity [11, 12], or changes in sea ice [13–15].

Marine bird prey in the North Pacific can be divided into three groups: forage fish and cephalopods, zooplankton, and benthic organisms [4]. All three are influenced by environmental drivers that affect phytoplankton productivity (e.g., [16]), which in turn influences the abundance of zooplankton like copepods and euphausiids (collectively, lower trophic levels [LTLs]), as well as productivity of the benthos [17]. Long-term changes in physical drivers are likely to impact LTLs, and thereby affect upper trophic levels. Such potential changes in biomass for LTLs can translate into changes in the biomass or distribution of pelagic fish and cephalopods, though the specific mechanisms for these changes can be difficult to quantify.

Commonly defined as the 2° C isotherm, the eastern Bering Sea “cold pool” varies in annual extent as much as 2° latitude from about 56°N in cold years to 58°N in warm years; Hermann et al. [18] note that while the cold pool shifts out of the southeastern Bering Sea in warm years, it normally persists in the northern Bering Sea. Along with this oscillation in annual extent is a trend of northward movement of the cold pool. The revised oscillating control hypothesis indicates that prolonged warm periods during early sea ice retreat diminishes pollock recruitment [19]. The cold pool determines the distribution of Arctic and sub-Arctic fishes and invertebrates, and research has shown that its northward migration by approximately 230 km over the last three decades has caused a shift in the ranges of numerous fish species [20]. Further warming will likely continue the trend of northward movement of some Arctic, cold-adapted organisms, with replacement by other sub-Arctic, warmer-water organisms [20].

Different seabird species vary in their responses to climate change, likely due to differing abilities to adapt to shifts in prey distribution and general forage availability; marine birds may face nutritional, demographic, or population stress (e.g., [21]), though analyses have only been completed on some species. Cury et al. [22] defined a concept of “one third for the birds” based on their finding that when prey abundance drops below a certain threshold, seabird breeding success declines significantly; their study provides an approximation of the minimum prey biomass required to sustain long-term seabird productivity. Dorresteijn et al. [8] suggested that a warming climate is likely to have a negative impact on planktivorous auklets (*Aethia* spp.) in the southeastern Bering Sea by decreasing food availability. Similarly, planktivorous auklets in other North Pacific studies link dietary shifts and productivity with changes in zooplankton availability [23–26]. However, zooplankton response to warm seas can vary, such as with calanoid copepods in the Southern Ocean, which appeared to be resilient to ocean warming [27]. Broadly, spatial patterns are heavily influenced by wind which plays a critical role in moving water masses that in turn influence water temperature, advective regimes, and zooplankton production. Hermann et al. [18] found from their models of the Bering Sea ecosystem (analyzed here) that large crustacean zooplankton production did not respond uniformly to climate change, rather it showed spatial variations with inner shelf areas increasing in biomass and outer shelf areas decreasing in response to warming temperatures; Coyle et al. [28] found that large zooplankton biomass in the Bering Sea declined in warm years.

At least one Arctic seabird species, the planktivorous Little Auk (*Alle alle*) has shown behavioral flexibility in diet and foraging distance that may allow them to overcome nutritional deficits and reproduce successfully [29], yet there is more conclusive evidence that other species are less adaptable to environmental changes based on recent mass mortality events in the North Pacific. Although mechanisms are unclear, during 2014 to 2017, extended periods of unusually warm waters in the North Pacific coincided with a number of mass mortality events of tens to hundreds of thousands of seabirds (e.g., Cassin’s auklets (*Ptychoramphus aleuticus*) [30]; Common Murres [31]; Tufted Puffins and others [32, 33].

In this study, we assessed the impacts of climate change projections and their potential effects on bird species throughout the Bering Sea and Aleutian Arc. First, we used three different climate models to assess climate vulnerability of marine birds and some components of their prey, examining seasonal and annual projections of six physical and biological variables. Second, we analyzed these variables at multiple scales, including in areas of known globally significant concentrations of marine birds. We analyzed the magnitude of projected change relative to baseline conditions for the region, as well as the agreement among models for exceeding the climate vulnerability threshold applied. Third, we incorporated these values into a coupled magnitude-agreement score and compared indices across areas and species.

**Fig 1:**
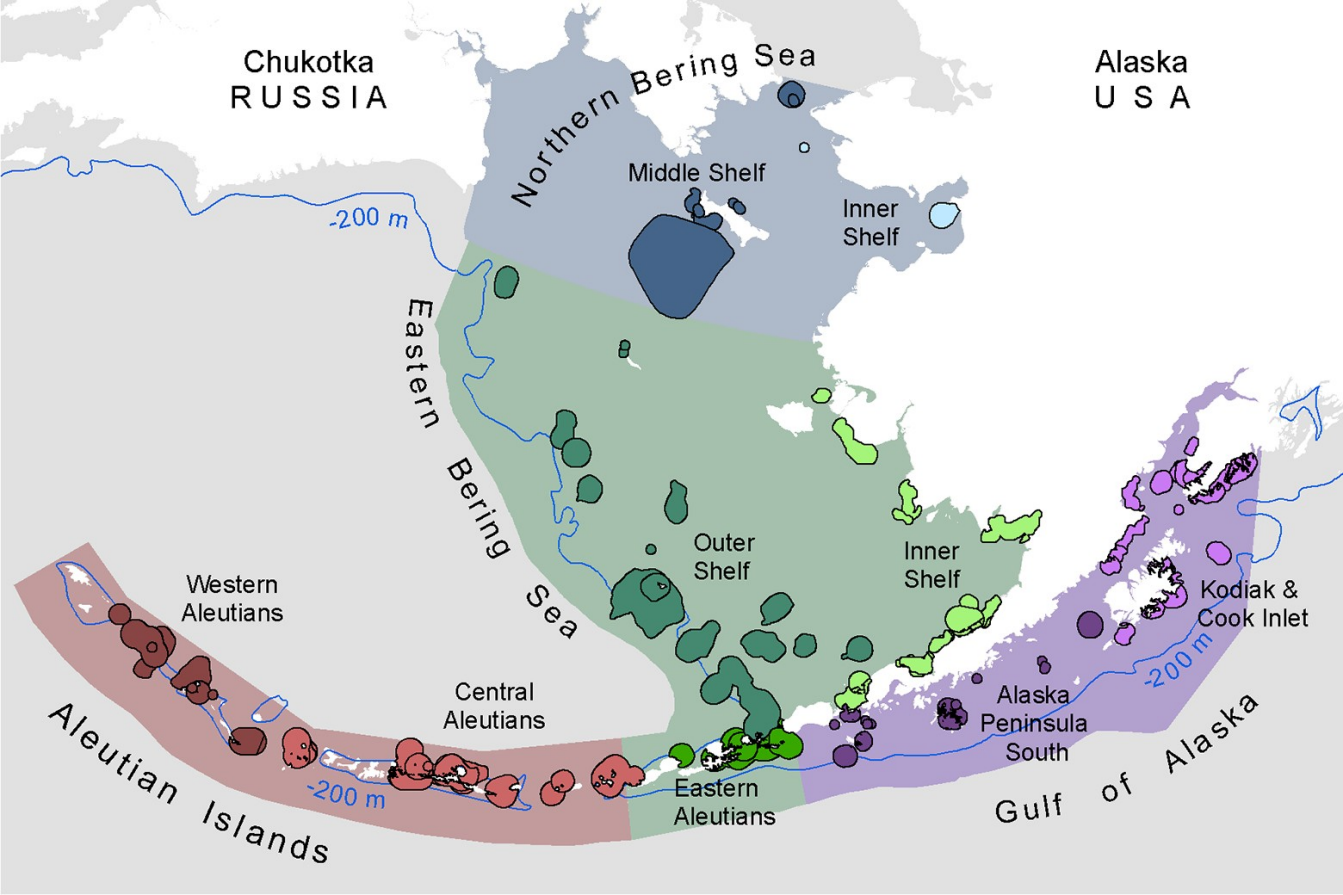
The Bering Sea and Aleutian Arc includes four Large Marine Ecosystems. Marine bird core areas assessed in this study are grouped into finer-scale marine ecoregions.

Multiple-scale assessment identifies differences in regional and local patterns that may yield useful information for management and conservation. Although averaging across biogeographic provinces may reveal the large-scale temperature regime or biomass availability, these broad geographic mean values do not elucidate small-scale patterns influenced by eddies or upwellings that shift prey biomass [34, 35]. Thus, our assessment placed local changes in context of the broader ecosystem variability to identify areas of vulnerability to climate change. This work examined climate vulnerability at multiple levels: 1) the study area; 2) Large Marine Ecosystems (LMEs), including the Gulf of Alaska (GOA), Aleutian Islands (AI), East Bering Sea (EBS), and Northern Bering—Chukchi Seas (NBS) [36]; 3) marine bird core areas grouped by LMEs and the marine ecoregions of Alaska [37]; and 4) marine bird species within four guilds (Fig 1).

## Methods

### Study area

The Bering Sea is a cold, deep-sea basin with an extensive continental shelf located between Alaska, USA and Chukotka, Russia. Our study area included the NBS from approximately 62° N to the Bering Strait), the EBS shelf (extending 480–725 km west from the Alaska mainland), a portion of the northern GOA shelf from Cook Inlet to Unimak Pass, and waters surrounding the AI to ~100 km offshore (Fig 1). We refer to these four regions collectively as the Bering Sea and Aleutian Arc.

The study area is known for its high biological productivity: over 80% of seabirds that breed in the USA nest in Alaska [38], and most of those (~20.3 million) nest on islands in the Bering Sea and along the Aleutian chain, roughly three times the number in the GOA [39]; diverse populations of marine mammals number in the millions [40]; and the region provides about half of USA fisheries production by weight, as well as the largest sockeye salmon fishery in the world [41, 42].

The Bering Sea and Aleutian Arc is a predominantly sub-Arctic, productive system that is influenced by sea ice, but less so than areas farther north in the Arctic Ocean. The system undergoes interdecadal warmer or cooler temperature regimes, known as the Pacific Decadal Oscillation (PDO), which has widespread impacts on natural systems such as marine fisheries [43]. Despite this natural oscillation, climate change is already evident in the Bering Sea ecosystem by a general warming trend across PDO cycles, lower sea ice concentrations, and smaller sea ice extent [3, 41].

Four LMEs within the study area have varying characteristics [36]. The northern GOA is productive for many species of pelagic and demersal fish, attracting pursuit-diving (e.g., puffins, *Fratercula* spp.) and surface-feeding seabirds (e.g., storm-petrels, *Oceanodroma* spp.) from numerous seabird colonies on islands and along the Alaska Peninsula coast. The AI hosts several million colonial nesting seabirds in a variety of nesting habitats, primarily crevice-nesting alcids (especially auklets) and burrow-nesting storm-petrels which depend on areas lacking native terrestrial predators.

The EBS is seasonally ice-covered only in the northern portion. The southern portion is a region of high productivity for both pelagic and demersal fishes, which are limited in their northern distribution by the cold pool [20, 44, 45]; the middle and inner shelves attract a high abundance of pursuit-diving birds such as murres (*Uria* spp.) [46, 47]. The outer shelf along the shelf break, also known as the “greenbelt” generates high zooplankton production, attracting abundant surface-foraging seabirds such as shearwaters (*Ardenna* spp.) [46, 47]. High benthic biomass on the inner shelf attracts globally significant abundances of benthic-feeding seaducks [48, 49]. Sea ice seasonally covers the continental shelf in the NBS; this is an area of high benthic biomass that attracts seaducks, and productive zooplankton blooms that attract millions of alcids, primarily auklets [17, 50].

### Climate and bird datasets

#### Important Bird Areas

We focused our analysis on known places of globally significant bird concentration using recognized Important Bird Areas (IBAs) [51], so that any significant changes that were found would be directly linked to identified important habitats for birds (Fig 1). IBAs often encompass overlapping core areas for multiple bird species. In order to relate potential changes in physical and biological variables to individual species’ foraging ecology, we used single-species core areas, which were a predecessor to integrated multi-species IBA boundaries, hereafter referred to as marine bird core areas.

We used the four LMEs [36] to summarize results of the analysis: GOA, AI, EBS, and NBS. We modified the EBS to exclude areas off of the continental shelf (deeper than 200 m) where no marine bird core areas were present and reported LME-focused results within the modified boundary only (Fig 1). For the vulnerability analysis, we associated each marine bird core area with the majority overlapping LME. For summarizing results, we associated each core area with an LME and a finer-scale marine ecoregion, which we based on the Marine Ecoregions of Alaska [37].

#### Ocean climate projection models

We assessed projected changes to the marine bird core areas using three downscaled, four-dimensional, coupled physical-biological models of ocean variables under the IPCC Fourth Assessment Report (AR4) emissions scenario A1B, from the National Oceanic and Atmospheric Administration’s Pacific Marine Environmental Laboratory [18, 52]. These projections were based on ocean climate models that pair a Regional Ocean Modeling System (ROMS) with climate model output extracted for the North Pacific from global climate models (GCMs). The downscaled variables have a spatial resolution of 10×10 km with 10 vertical layers (e.g, biomass of euphausiids at different depths in the water column). At this resolution, the models are reasonably well matched for assessing units the general size of IBA core areas; some of the smallest core areas were removed to avoid overpredicting from these broadly generated models. The models are not meant to predictively reproduce measurements from fine-scale field studies which is a limitation in their application to projecting precise forage changes and population effects.

Hermann et al. [52] describe these downscaled models and compare projections from the Coupled Global Climate Model (CGCM) t47 grid, from the Canadian Centre for Climate Modelling and Analysis to the Coordinated Ocean-Ice Reference Experiments (CORE) hindcast climate model [53]. Hermann et al. [18] then developed two other models that we analyzed: the Max Planck Institute’s ECHAM4+HOPE-G (ECHO-G) model, and the Model for Interdisciplinary Research on Climate (MIROC), 3.2-Medres, developed by a consortium of agencies in Japan. According to Hermann et al. [18], the CGCM model realization showed the greatest warming over time; MIROC showed intermediate warming, and ECHO-G showed the least warming (all under the SRES A1B emissions scenario). The authors emphasized that these three model realizations constituted a small ensemble, and captured only a portion of the variability of all realizations carried out under AR4. The models were acquired as raster layers from the Alaska Ocean Observing System’s Arctic Data Integration Portal (http://portal.aoos.org/arctic.php).

The models developed by Hermann et al. [52] represent a sub-Arctic ecosystem including multiple physical, nutrient, phytoplankton, and zooplankton components; the base model was coupled with ice biology and benthic biology modules at the sea surface and sea floor, respectively [54]. From the 14 available modeled variables, we selected 2 physical variables that drive the marine ecosystem (seawater temperature [SWT] and sea ice concentration) and 3 biological forage variables that include LTL species found in the diets of the species included in this study (large copepods [represented by *Neocalanus* and other similar spp.], euphausiids, and benthic infauna; Table 1). Cold pool dynamics were integrated into the models and are incorporated into our assessment through analysis of SWT data, but otherwise modeled changes to the cold pool are not specifically addressed in this paper. Model output tracks the biomass of large copepods and euphausiids measured as mg C/m^3^ for various levels of the water column; these LTL variables are exclusive of “small microzooplankton (heterotrophic nanoflagellates, ciliates and medium dinoflagellates), large microzooplankton (large heterotrophic dinoflagellates), and small coastal copepods (represented by Pseudocalanus spp.)” [55]. The model tracks the biomass of benthic infauna as mg C /m^2^ which is a single layer model with no vertical resolution; the benthic variable “represents the dominant infauna groups in the Bering Sea that live wholly or partly within the sediment, i.e., bivalves, amphipods, and polychaetes” [54].

**Table 1:** Physical and biologic variables and depth classes considered in the vulnerability assessment, from downscaled ocean climate models by Hermann et al. [52].

**Physical Variables:**
Seawater temperature (SWT; °C), shallow (0–60 m)
Seawater temperature (SWT; °C), deep (75–200 m)
Sea ice (% ice fraction area), surface only
**Biological Variables:**
Large copepods (mg C/m^3^), shallow (0–60 m)
Euphausiids (mg C/m^3^), shallow (0–60 m)
Benthic infauna (mg C/m^2^), bottom only

We were not able to assess fish, cephalopods, or other forage resources directly in this study due to lack of data availability for projected fish biomass. Four of the selected variables were assessed by combining multiple depth classes. SWT was summarized in two categories: shallow waters (0–60 m depth) and deep waters (75–200 m depth). Large copepods and euphausiids were summarized for shallow waters only (0–60 m). Sea ice cover and benthic infauna, at the ocean surface and bottom respectively, did not have depth classes. Sea ice projection data layers, which covered the entire study area inclusive of non-ice regions, were clipped to the 2006–2015 median maximum ice extent from Knight et al. [56].

### Change analysis for physical and biological variables

We ran within-model analyses to look at changes between modeled recent baseline conditions and future conditions for the six variables. Like Hermann et al. [18], we compared the recent 10-year time period at the beginning of the model (2003–2012) to a future 10-year time period at the end of the model (2030–2039), for each of the CGCM, MIROC, and ECHO-G models. For SWT, we used all available year-round data to calculate change in annual temperature. For sea ice, we used only spring season data since spring ice extent and concentration greatly influences ocean productivity during the ice-free season. We assessed the forage variables seasonally to match the primary season each marine bird core area was occupied, breaking the data down into winter (December through February), spring (March through May), summer (June through August), or fall (September through November). Introducing seasonality into the assessment captured not only the change in average biomass but the timing of that biomass in relation to when birds rely on it most.

We used the NetCDF Operator Suite [57] to statistically summarize the time-series data for each model. Mean values for each 100 km^2^ raster cell were calculated by computing the average from the weekly time slices of available input data from each climate model for the recent and future time periods. Our code is published in an open-access iPython script available at https://github.com/wckoeppen/bering-seabird-vulnerability, available to others to adapt to their own analyses. We also calculated area-wide means and standard deviations (SD) geographically for each of four reference LMEs, used in a later step to normalize data values for calculations of projected LME changes. For the analysis of core areas, we calculated SD over a moving window of the nearest 2500 cells with data from the core area centroid.

#### Relating core areas to species forage variables

Our study focused on marine birds within four guilds: petrels, seaducks, alcids, and gulls/terns. We developed a table of foraging types for the species in order to match core areas with the relevant forage variables (S1 Table), based on published species accounts [58] and expert knowledge. Typically, marine birds use multiple types of prey to meet their foraging needs [59]. Prey selection can vary with relative abundance as well as the energetic value of different prey types [60], and geographically or interannually within the same species [61, 62]. As noted in the table, many species in our study rely on fish, cephalopods, and other mesozooplankton as a main prey source; however, these were not available as projected forage variables. They are marked here (S1 Table) for completeness and to convey an unaddressed element of this study in determining climate vulnerability. Marine species that rely solely on fish as their primary prey (e.g., puffins, loons [*Gavia* spp.], cormorants [*Phalacrocorax* spp.]) were removed from the analysis, as well as nearshore vegetation specialists (e.g., geese [Anatidae]).

We only included results for marine bird core areas with a total spatial extent of greater than 300 km^2^ (minimum 3 or more raster cells of data at each available depth class). Within the 138 core areas evaluated (constituting 66 marine IBAs), there were 26 species assessed (those present in globally significant abundance per IBA criteria). These included a mix of colony, nearshore, and pelagic IBAs. There were 25 core areas in winter, 15 in spring, 88 in summer, and 10 in fall.

#### Calculating standard deviation (SD) change values

Changes in temperature, sea ice cover, and forage biomass are contextualized by comparing those values to the baseline (recent average) condition of the region; a small absolute change for one variable may be much more significant than the same small change in another variable. To standardize change values, we indexed the amount of projected change for each variable within each marine bird core area by finding the projected change from the baseline condition within each raster cell. Cell values within the minimum bounding rectangle of each marine bird core area were then averaged. To derive the standard deviation (SD) change value, also referred to here as the magnitude of change, we calculated the difference between the mean of the core area cells in time 1 (t_1_) and time 2 (t_2_), normalized by the SD of the reference region in t_1_. The SD of each reference region is the SD across all raster cells within the region boundary where each cell represents the mean value during t_1_. This simplified down to subtracting the recent 2003–2012 core area mean (μ_t1_) from the future 2030–2039 core area mean (μ_t2_), divided by the SD of the reference region for the recent 2003–2012 time period (SD_t1_), summarized in the following equation:

$$SD\;change\;value=(\mu_{t2}-\mu_{t1})÷{SD}_{t1}$$

Using this approach allowed us to translate data with disparate units (e.g., degrees C, mg C) to a comparable, relative scale to facilitate assessment across variables for each region or core area.

#### Interpreting vulnerability based on SD change values

Significant decreases in physical and biological parameters were considered an indication of vulnerability to climate change, with the exception of SWT for which significant increase was of concern. To identify climate-vulnerable core areas, we applied a threshold based on the concept of “one-third for the birds.” Cury et al. [22] found that prey abundance of one-third of the maximum long-term prey biomass is the threshold above which seabird productivity is sustained over the long term. Like our study, they normalized and expressed data by the number of SD from the mean to enable comparisons across species and ecosystems. Their change-point analysis identified a threshold of ~0.10 SD, the most likely point at which the slope of breeding success changed in relation to prey abundance. In our study, areas with SD change values greater than 0, or those changing less than -0.1 were categorized as low vulnerability (again, except for SWT which was categorized in the opposite direction), and were not classified as climate vulnerable. Theoretically, a change of 1 or more SDs would indicate a shift outside of the central values of the normal distribution which, given the relatively short time frame of this study (<40 years), could indicate a rapid oncoming state change in the system. To take a look at extreme values, we categorized a change of 1 or more SDs as having a high level of climate vulnerability (Table 2).

**Table 2:** SD change values categorized into levels of vulnerability for climate change.

SD Change Value	Vulnerability Category^a^	Classification
≥ 0; -0.01 to -0.09	Low	Dismissed from further discussion
-0.1 to -0.99	Moderate	Climate
≤ -1.000	High	vulnerable

^a^ SWT SD change values were categorized in the opposite direction, with positive values indicating vulnerability.

Next, for each marine bird core area, the physical variables and the applicable prey variables were assessed across the three models to identify vulnerability agreement and magnitude of change. Vulnerability agreement was calculated as number of models (out of a possible three) indicating climate vulnerability (moderate or high categories), summed across core areas, divided by the total possible agreement score (the number of core areas times three). Magnitude was the average of SD change values resulting from the three models, averaged across core areas. To combine the two terms into a final ranking, we multiplied the magnitude by the vulnerability agreement to calculate the magnitude-agreement score, an indication of the relative amount of projected change mediated by the relative vulnerability agreement among the model projections.

## Results

### Study area climate projections

We first examined overall trends across the study area to characterize broad-scale changes indicated by the climate projections. The combined model results indicate that annual shallow SWT is expected to rise by 0.55 °C (range +0.20 –+0.82) by 2040 (Table 3), smaller than the 1+ °C shift in most-likely temperature reported for these models by Hermann et al. [18]. In shallow water, CGCM projected the greatest warming of the models for winter (+0.95 °C), spring (+0.69 °C), and fall (+0.81 °C), while MIROC showed the greatest summer warming (+0.88 °C); CGCM showed the greatest warming (+0.75 °C) of the models for deep water. ECHO-G most often indicated the least warming; yet ECHO-G and MIROC tied for deep SWT annual change (Table 3). The combined models indicated an annual sea ice cover loss of 3–9% by 2040, with spring rates of loss ranging from 9–14% within the seasonally ice-covered portion of the study area (Table 3).

**Table 3:** Projected mean change across the study area between recent (2003–2012) and future (2030–2039) time periods.

**Shallow seawater temperature (°C)**	**Winter**	**Spring**	**Summer**	**Fall**	**Annual**
MIROC	+0.33	+0.50	+0.88	+0.77	+0.63
CGCM	+0.95	+0.69	+0.81	+0.81	+0.82
ECHO-G	+0.02	+0.26	+0.29	+0.30	+0.20
3-Model Average	+0.43	+0.48	+0.66	+0.63	+0.55
**Deep seawater temperature (°C)**	**Winter**	**Spring**	**Summer**	**Fall**	**Annual**
MIROC	+0.37	+0.30	+0.34	+0.39	+0.34
CGCM	+0.75	+0.71	+0.74	+0.80	+0.75
ECHO-G	+0.24	+0.33	+0.40	+0.35	+0.34
3-Model Average	+0.45	+0.45	+0.49	+0.51	+0.48
**Sea ice concentration (%)**	**Winter**	**Spring**	**Summer**	**Fall**	**Annual**
MIROC	-10.79	-13.61	-7.16	-4.20	-8.96
CGCM	-13.02	-8.73	-4.33	-4.59	-7.54
ECHO-G	+1.16	-8.98	-3.76	-1.28	-2.69
3-Model Average	-7.55	-10.44	-5.08	-3.35	-6.40
**Large copepods (mg C / m**^**3**^**)**	**Winter**	**Spring**	**Summer**	**Fall**	**Annual**
MIROC	-0.01	-0.07	+0.11	+0.04	+0.02
CGCM	-0.02	+0.01	+0.12	-0.03	+0.02
ECHO-G	0.00	+0.06	+0.03	+0.01	+0.03
3-Model Average	-0.01	0.00	+0.08	+0.01	+0.02
**Euphausiids (mg C / m**^**3**^**)**	**Winter**	**Spring**	**Summer**	**Fall**	**Annual**
MIROC	-0.07	-0.11	+0.27	-0.02	+0.01
CGCM	-0.16	+0.05	+0.27	-0.11	+0.01
ECHO-G	-0.03	+0.17	0.00	+0.03	+0.06
3-Model Average	-0.09	+0.04	+0.18	-0.03	+0.03
**Benthic infauna (mg C / m**^**2**^**)**	**Winter**	**Spring**	**Summer**	**Fall**	**Annual**
MIROC	+77.00	+64.62	+235.71	+219.60	+152.82
CGCM	-111.14	-91.82	+83.61	-23.85	-37.75
ECHO-G	-36.40	-6.36	+62.62	+5.36	+29.11
3-Model Average	-23.52	-11.19	+127.31	+67.04	+48.06

**Fig 2:**
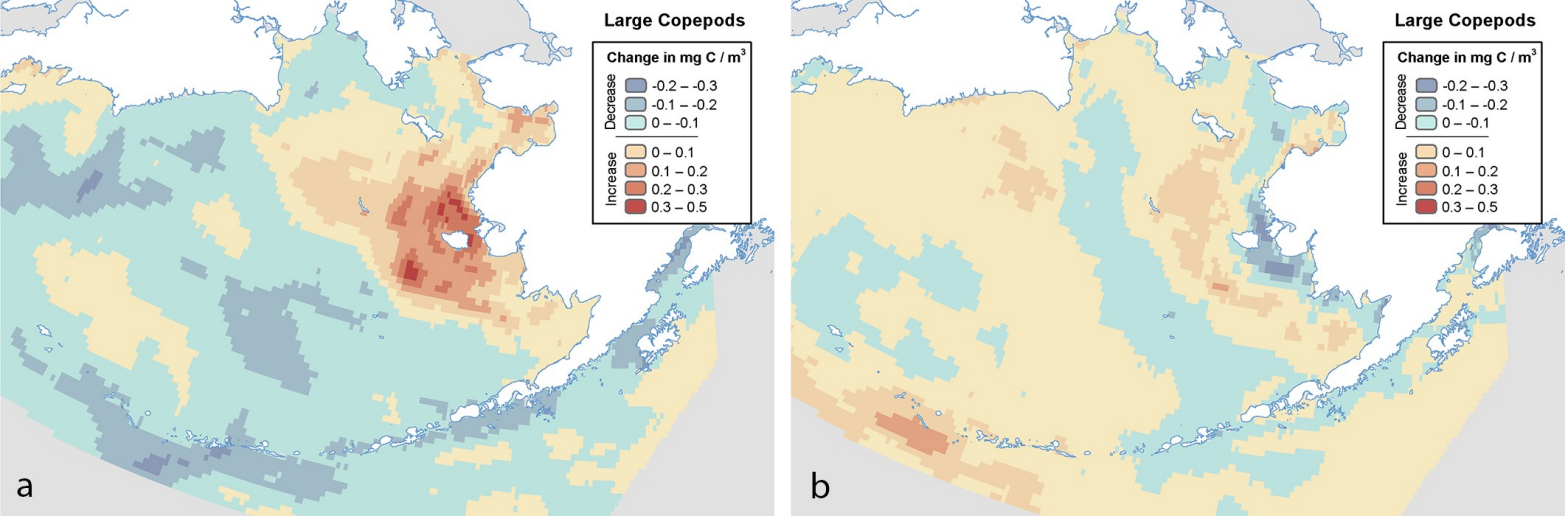
Projected change in large copepod biomass between 2003–2012 and 2030–2039 for the (a) MIROC and (b) CGCM models. Note that the same geographic location may show increasing biomass in one model but decreasing biomass in the other.

All three models were consistent in their projection of increased SWT and decreased sea ice, with the exception the ECHO-G winter value showing a 1% increase. LTLs on average varied little from baseline conditions and values varied in direction by season and by model; consistency was observed in the winter decrease of LTLs and the summer increase for all three forage variables. Inconsistency in forage values is unsurprising at this scale, given that study-area-wide values were averaged across multiple water masses and geographic areas that have high variability; evaluation of these parameters at finer scales is more appropriate. However, even at finer scales, the models were spatially inconsistent, with disagreement among results in the same geographic location (Fig 2, S2–S4 Tables).

To contextualize these data we summarized the SD change value for each variable, by model and season, as well as the three-model average (Table 4). The largest projected changes are in SWT, with shallow water warming by as much as +0.37 SD in spring or +0.33 SD annually. Projected sea ice cover changes were greatest among all variables and were significant for all models in all seasons, except singularly for ECHO-G winter estimates. At the study area scale, few forage variables were projected to have significant decline, but euphausiids stand out most notably for negative changes projected by the MIROC and CGCM models.

**Table 4:** Projected magnitude of change, measured as the change in SD from the recent (2003–2012) study area mean to the future (2030–2039) time period, for three ocean climate models. Bold values exceed the climate vulnerability threshold (+/- 0.1 SD) applied in this study.

**Shallow seawater temperature (°C),**	**Winter**	**Spring**	**Summer**	**Fall**	**Annual**
MIROC	**+0.15**	**+0.22**	**+0.37**	**+0.32**	**+0.28**
CGCM	**+0.38**	**+0.29**	**+0.31**	**+0.29**	**+0.33**
ECHO-G	+0.01	**+0.12**	**+0.12**	**+0.13**	+0.09
3-Model Average	**+0.18**	**+0.21**	**+0.27**	**+0.25**	**+0.23**
**Deep seawater temperature (°C)**	**Winter**	**Spring**	**Summer**	**Fall**	**Annual**
MIROC	**+0.21**	**+0.16**	**+0.18**	**+0.22**	**+0.19**
CGCM	**+0.36**	**+0.32**	**+0.33**	**+0.39**	**+0.35**
ECHO-G	**+0.10**	**+0.13**	**+0.15**	**+0.15**	**+0.14**
3-Model Average	**+0.22**	**+0.20**	**+0.22**	**+0.25**	**+0.23**
**Sea ice concentration (%)**	**Winter**	**Spring**	**Summer**	**Fall**	**Annual**
MIROC	**-0.39**	**-0.78**	**-0.72**	**-0.48**	**-0.65**
CGCM	**-0.54**	**-0.41**	**-0.42**	**-0.36**	**-0.50**
ECHO-G	+0.05	**-0.41**	**-0.49**	**-0.17**	**-0.20**
3-Model Average	**-0.29**	**-0.54**	**-0.54**	**-0.34**	**-0.45**
**Large copepods (mg C / m**^**3**^**)**	**Winter**	**Spring**	**Summer**	**Fall**	**Annual**
MIROC	-0.07	**-0.12**	+0.25	+0.06	+0.06
CGCM	**-0.16**	+0.04	+0.21	-0.05	+0.07
ECHO-G	+0.01	+0.18	+0.07	+0.02	+0.13
3-Model Average	-0.07	+0.04	+0.17	+0.01	+0.09
**Euphausiids (mg C / m**^**3**^**)**	**Winter**	**Spring**	**Summer**	**Fall**	**Annual**
MIROC	**-0.10**	-0.09	+0.23	-0.02	+0.02
CGCM	**-0.34**	+0.05	+0.24	**-0.12**	+0.03
ECHO-G	-0.04	+0.17	0.00	+0.04	+0.10
3-Model Average	**-0.16**	+0.05	+0.16	-0.03	+0.05
**Benthic infauna (mg C / m**^**2**^**)**	**Winter**	**Spring**	**Summer**	**Fall**	**Annual**
MIROC	+0.05	+0.06	+0.13	+0.11	+0.10
CGCM	-0.07	-0.09	+0.04	-0.01	-0.02
ECHO-G	-0.02	-0.01	+0.03	0.00	+0.02
3-Model Average	-0.01	-0.01	+0.07	+0.04	+0.03

### Climate projections across LMEs

In the next scale down in the analysis, we averaged results of the three models across the extent of the four LMEs (Table 5). As an annual average, the EBS is projected to have the most warming, at 0.77 °C for shallow water, followed by the GOA, AI, and NBS. Sea ice is most concentrated in the NBS, and will decrease the most in the EBS (-7.4%; Table 5). At this scale, the biomass of large copepods, euphausiids, and benthic infauna are projected to increase in all LMEs but the GOA. The largest increases were in the EBS for large copepods and the NBS for euphausiids and benthic infauna (Table 5). This scale of analysis begins to address regional effects, but these values may be misleading as they do not elucidate the finer scale spatial heterogeneity of resources.

**Table 5:** Projected ocean conditions for the recent (2003–2012) and future (2030–2039) time periods averaged across models, summarized by Large Marine Ecosystems.

	Gulf of Alaska		Aleutian Islands		Eastern Bering Sea		Northern Bering Sea	
Variable	Recent mean ± SD	Projected change	Recent mean ± SD	Projected change	Recent mean ± SD	Projected change	Recent mean ± SD	Projected change
Shallow seawater temperature (°C)	5.07 ± 1.15	+0.56	5.55 ± 0.41	+0.33	2.52 ± 1.38	+0.77	0.03 ±0.64	+0.31
Deep seawater temperature (°C)	5.61 ± 1.18	+0.56	5.84 ± 0.63	+0.31	3.73 ± 1.72	+0.68	0.20 ±1.00	+0.10
Sea ice cover (%)	na	na	na	na	27.4 ± 10.6	-7.4	49.0 ±7.2	-5.5
Large copepods (mg C / m^3^)	0.386 ± 0.372	-0.014	0.342 ± 0.118	+0.006	0.561 ± 0.252	+0.051	0.345 ± 0.234	+0.028
Euphausiids (mg C / m^3^)	2.322 ± 0.926	-0.068	2.298 ± 0.717	+0.003	2.515 ± 0.497	+0.044	1.901 ± 0.321	+0.115
Benthic infauna (mg C / m^2^)	1744 ± 2044	-63	41 ± 141	+5	2726 ± 1224	+19	3040 ± 684	+195

We summarized the SD change values for the data presented in Table 5. Even though the EBS and GOA are projected to have the greatest warming, the AI had the greatest magnitude of change relative to its baseline conditions (Table 6). Overall, the EBS is projected to see the largest magnitude increase in large copepod biomass; NBS is projected to see the largest increases for euphausiids and benthic infaunal biomass (Table 6).

**Table 6:** Annual projected magnitude of change between 2003–2012 and 2030–2039 averaged across models, by Large Marine Ecosystems. Bold values exceed the climate vulnerability threshold (+/- 0.1 SD) applied in this study.

Variable	Gulf of Alaska	Aleutian Islands	Eastern Bering Sea	Northern Bering Sea
Shallow seawater temperature	**+0.49**	**+0.80**	**+0.56**	**+0.48**
Deep seawater temperature	**+0.47**	**+0.49**	**+0.40**	**+0.10**
Sea ice cover	na	na	**-0.70**	**-0.76**
Large copepods	-0.04	+0.05	+0.20	+0.12
Euphausiids	-0.07	<+0.01	+0.09	+0.36
Benthic infauna	-0.03	+0.04	+0.02	+0.29

### Climate projections for marine bird core areas

Scaling the analysis down again, we compared each marine bird core area to the reference condition of the nearest 2500 cells with data. With this step down in scale, SD change values reflect localized patterns of change versus system-wide or region-wide averages that mask highs and lows. This step allowed us to identify specific places and species for which significant change is projected.

**Fig 3:**
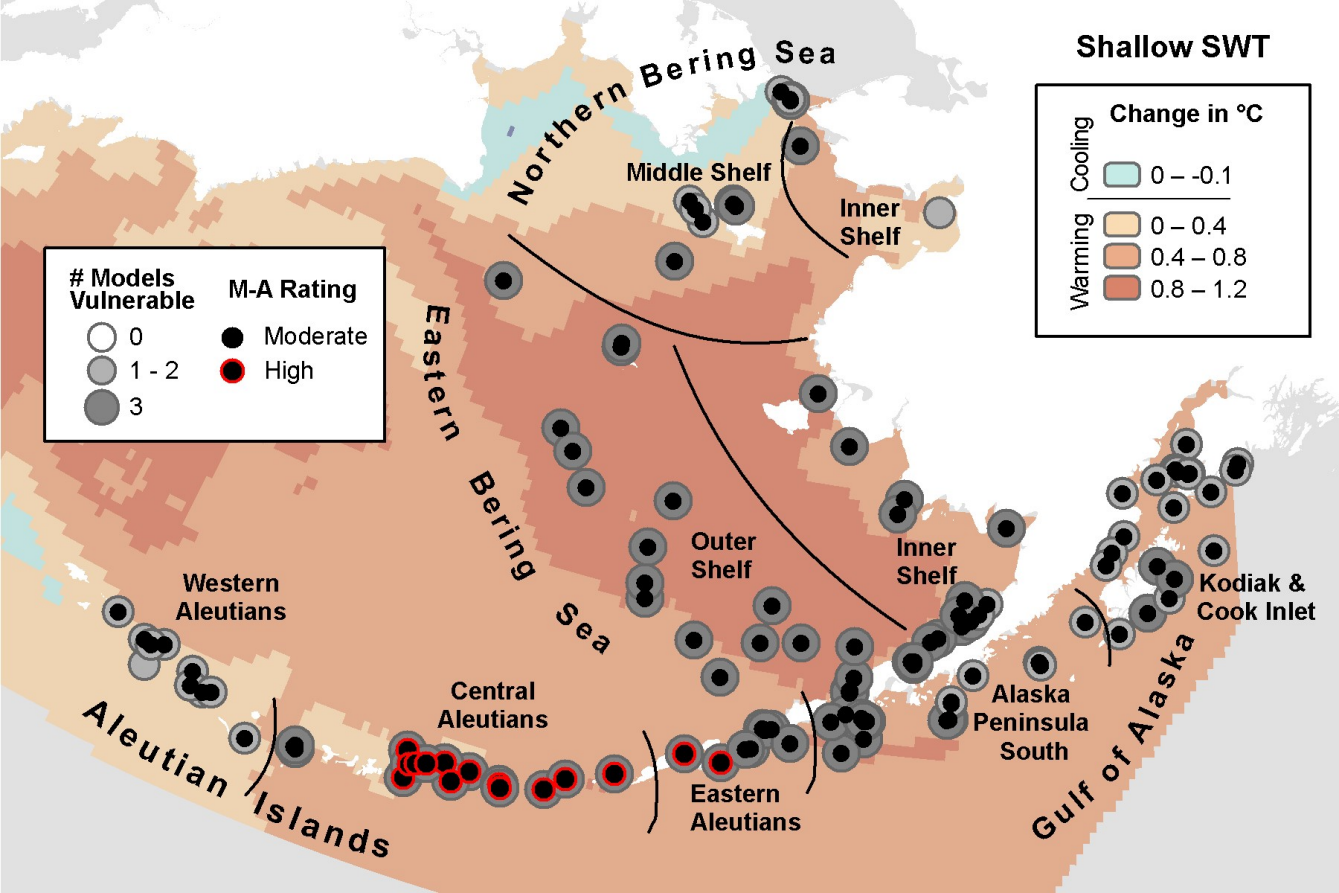
Three-model average change in shallow seawater temperature with marine bird core areas showing the number of models indicating climate vulnerability and the overall vulnerability rating based on the magnitude-agreement score.

**Fig 4:**
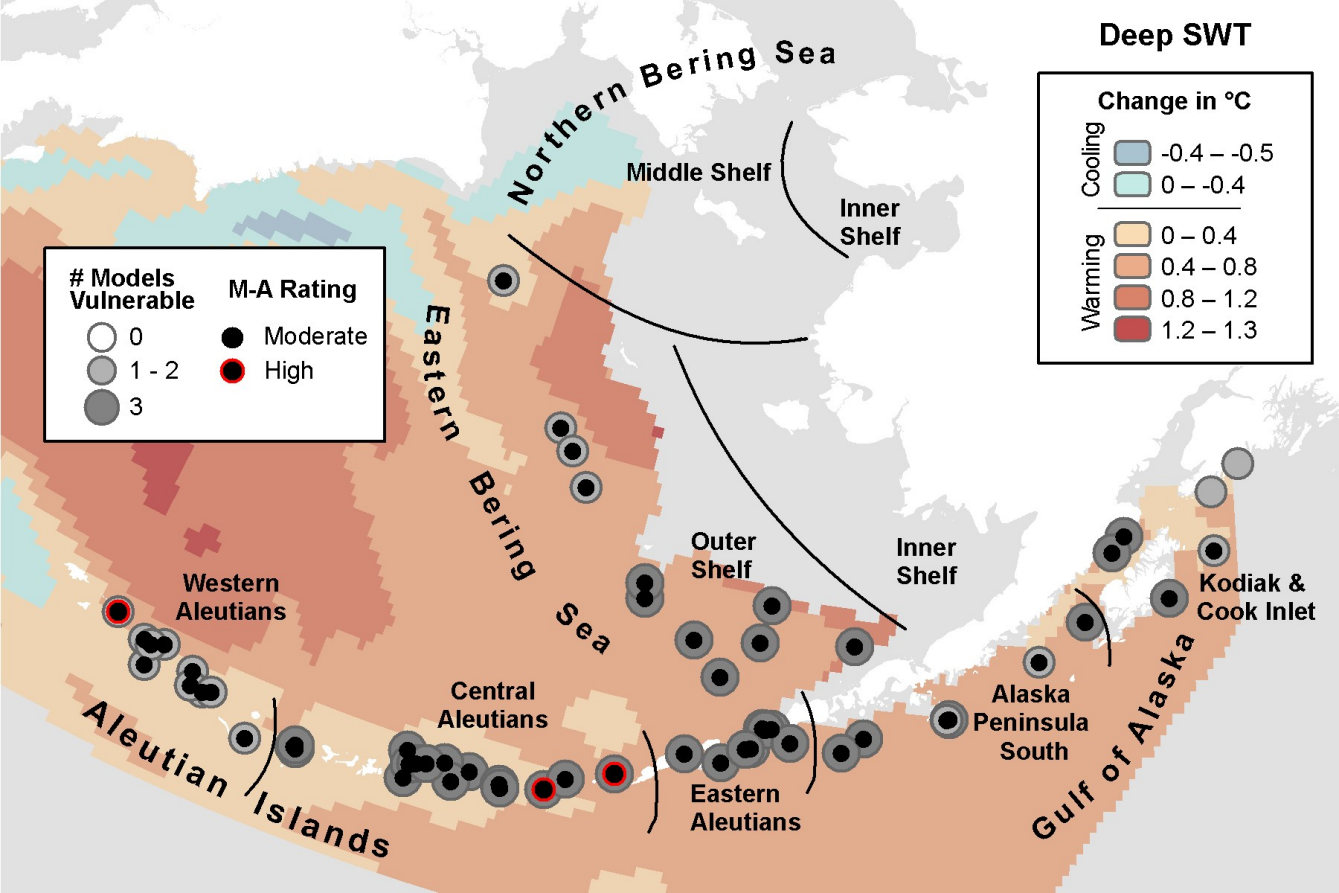
Three-model average change in deep seawater temperature with marine bird core areas showing the number of models indicating climate vulnerability and the overall vulnerability rating based on the magnitude-agreement score.

**Fig 5:**
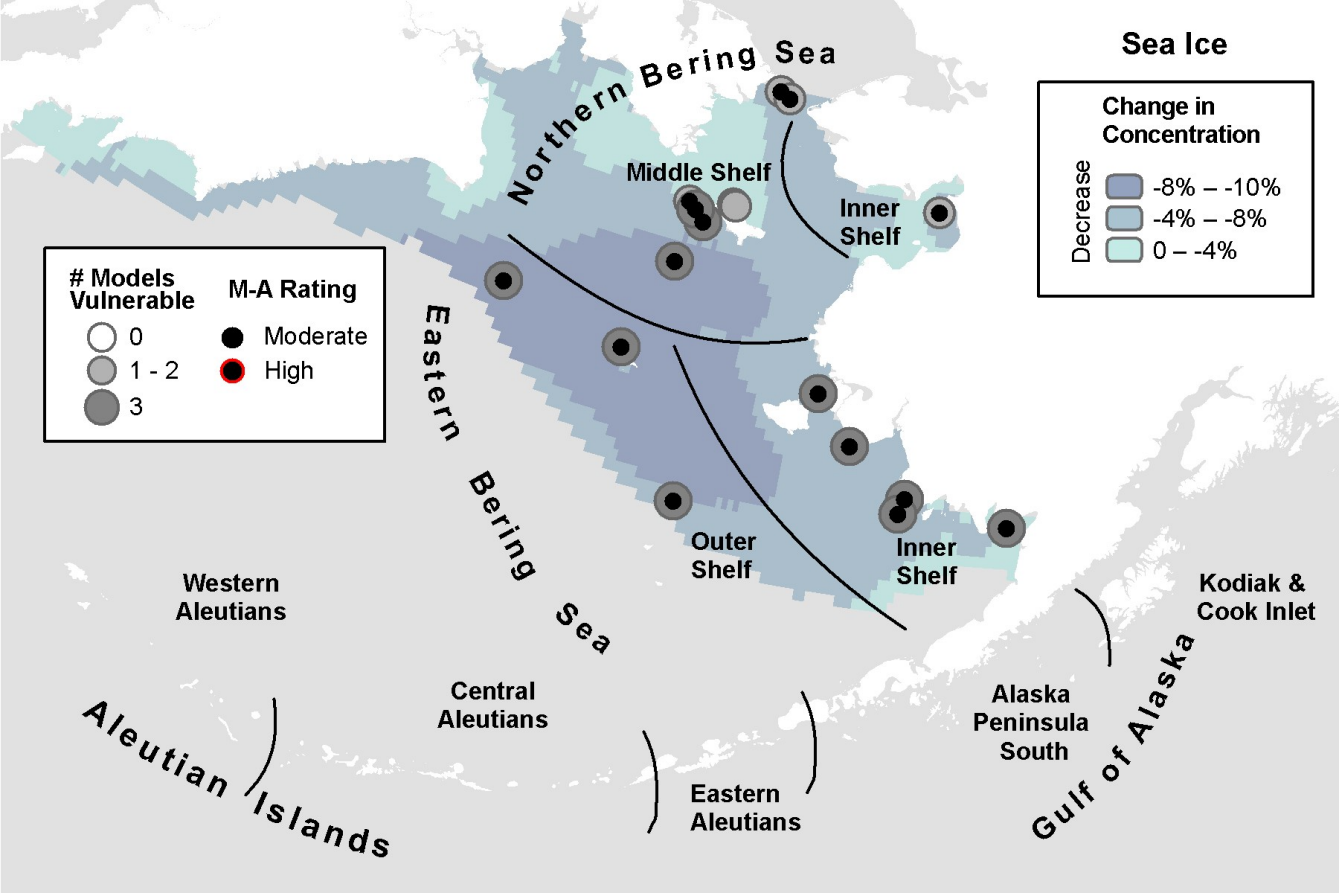
Three-model average change in sea ice concentration with marine bird core areas showing the number of models indicating climate vulnerability and the overall vulnerability rating based on the magnitude-agreement score.

**Fig 6:**
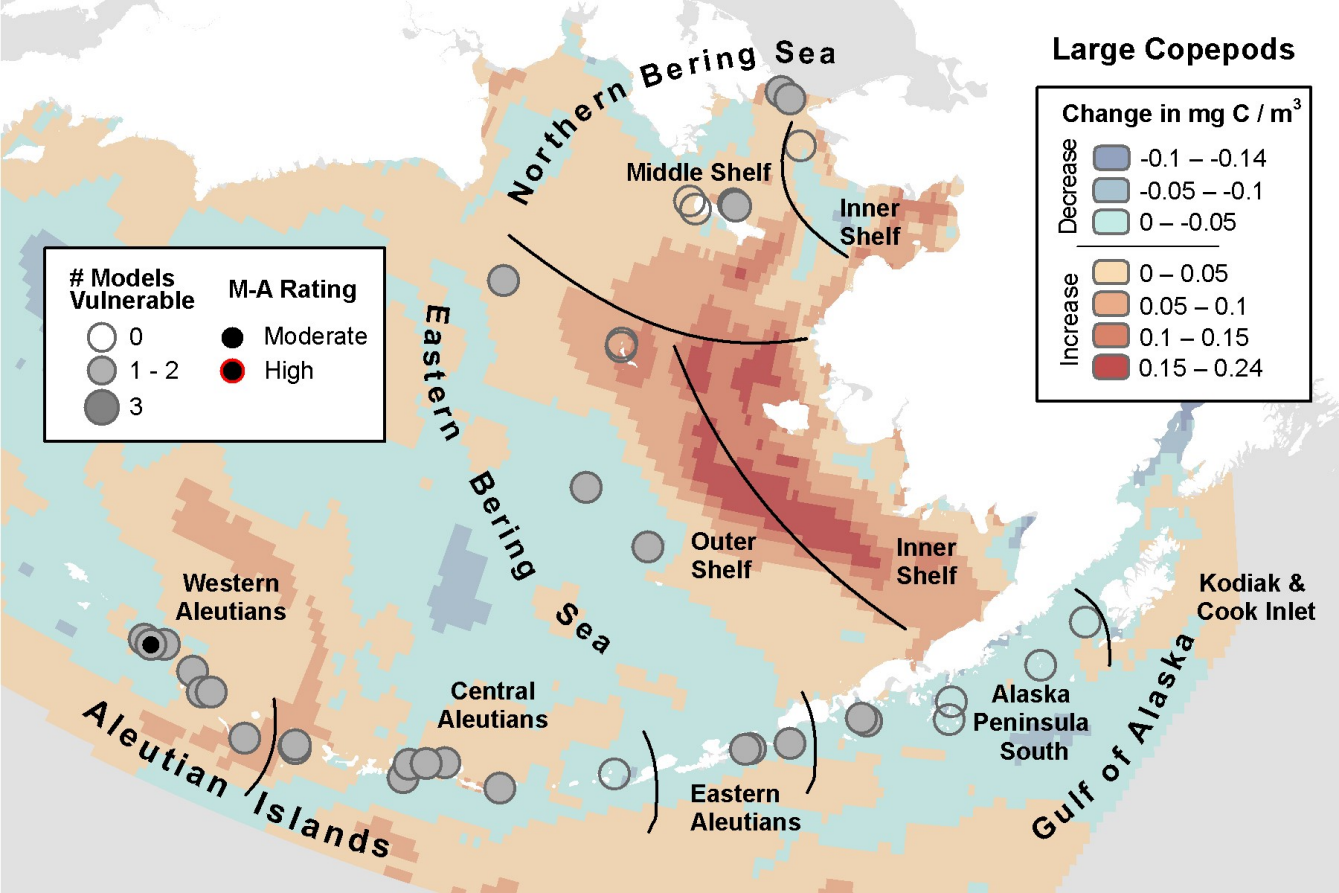
Three-model average change in large copepod biomass with marine bird core areas showing the number of models indicating climate vulnerability and the overall vulnerability rating based on the magnitude-agreement score.

**Fig 7:**
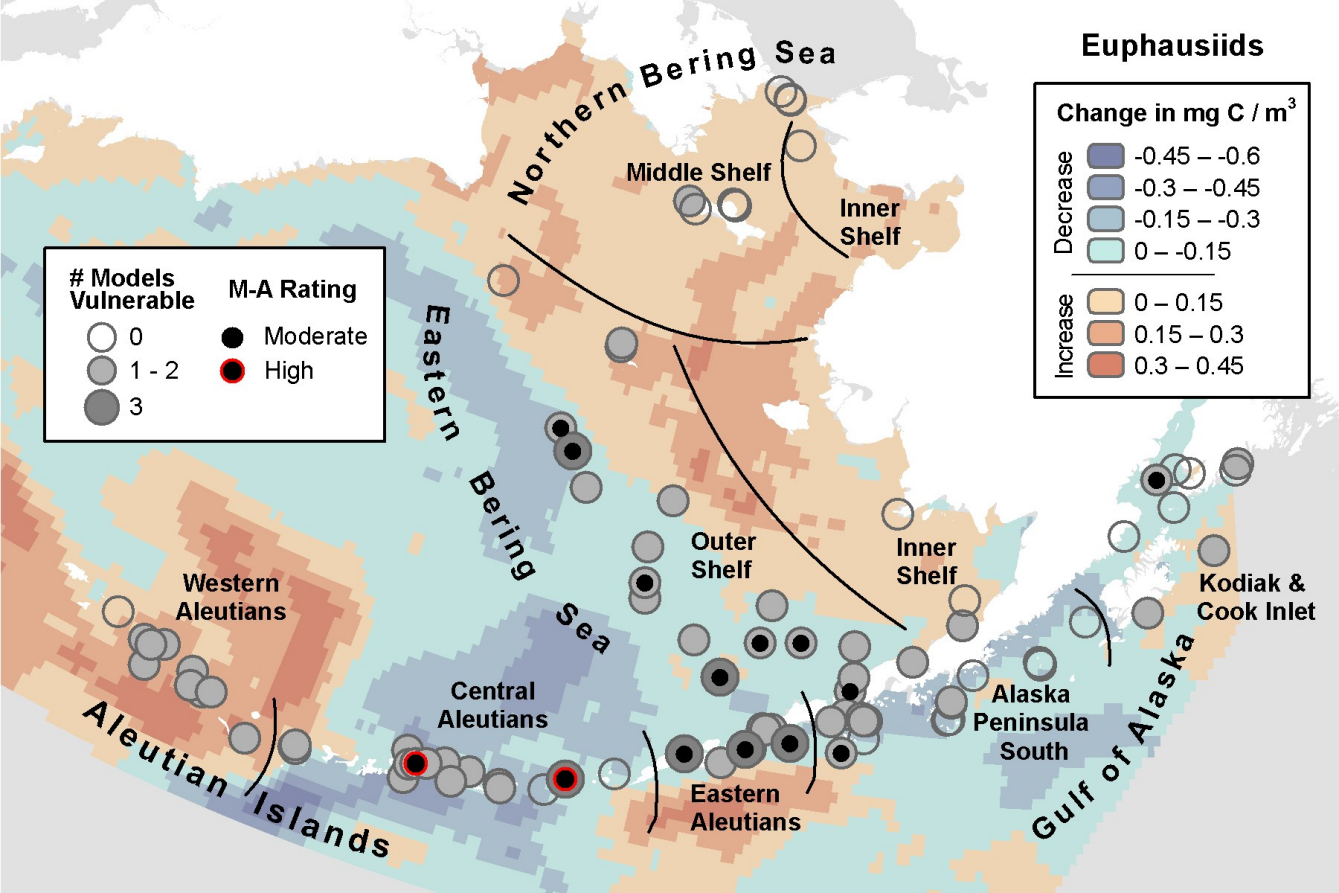
Three-model average change in euphausiid biomass with marine bird core areas showing the number of models indicating climate vulnerability and the overall vulnerability rating based on the magnitude-agreement score.

**Fig 8:**
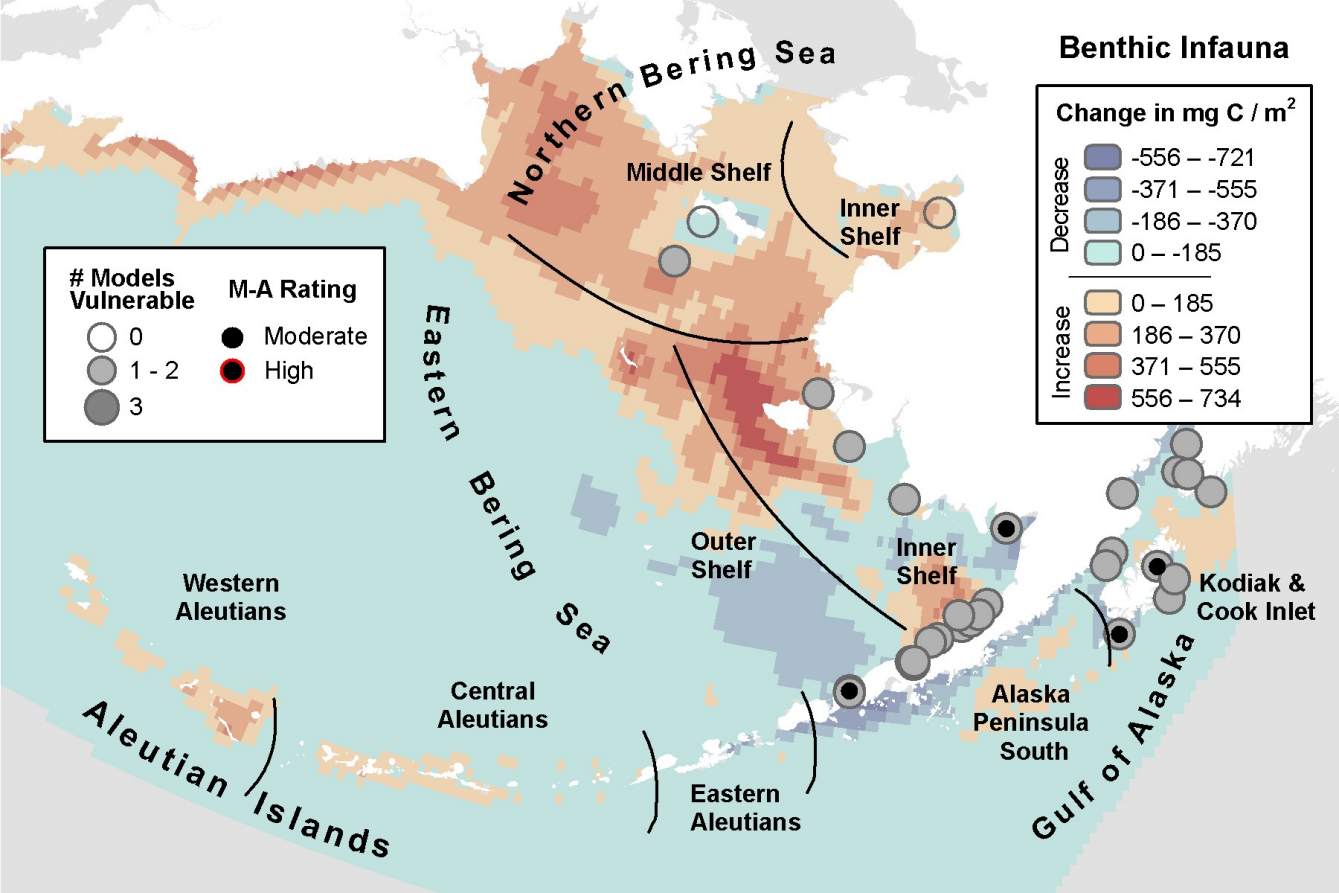
Three-model average change in benthic infaunal biomass with marine bird core areas showing the number of models indicating climate vulnerability and the overall vulnerability rating based on the magnitude-agreement score.

We mapped the three-model average change for each of the variables along with the number of models indicating climate vulnerability for each core area, as well as the areas with moderate or high vulnerability based on the magnitude-agreement score (Figs 3–8). For each variable we describe notable spatial patterns among the three models and for the magnitude-agreement score combining models. Additional detail, including a summary of projected magnitude of change, vulnerability agreement among models, and magnitude-agreement scores by core area (S2–S4 Tables) and maps of individual model results (S1 Figs) can be found in the supplementary materials.

#### Shallow seawater temperature (SWT)

Shallow SWT is projected to increase across nearly the entirety of the study area in all three models. The MIROC model identified all 138 core areas as moderate or high vulnerability (Table 7). Of those, 39 core areas for 14 species within 16 IBAs were in the high vulnerability category, primarily throughout the AI (S1 Figs). The CGCM model identified 120 of 138 core areas as displaying moderate vulnerability. Thirty-eight areas for 14 species within 18 IBAs rated in the high vulnerability category, located in the Central Aleutians, the Eastern Aleutians, EBS Outer Shelf, and the Alaska Peninsula South. ECHO-G identified 110 core areas as moderate vulnerability, with 10 core areas for 5 species in 4 IBAs rated in the high vulnerability category in the Central Aleutians.

**Table 7:** Summary of the number of species core areas projected to have moderate (> +0.1 SD change) or high (> +1 SD change) climate vulnerability due to shallow seawater temperature increase. Data presented are for each of three climate models analyzed plus the magnitude-agreement (M-A) score summarizing average SD change and agreement across models.

		MIROC	GCGM	ECHO-G	M-A Score	Total Core Areas
Guild	Species	Mod, High	Mod, High	Mod, High	Mod, High	
Petrels	Fork-tailed Storm-Petrel	4, 2	3, 1	4, 0	4, 1	4
	Leach’s Storm-Petrel	2, 1	1, 0	2, 0	2, 0	2
	Northern Fulmar	6, 3	6, 3	5, 3	6, 3	6
	Sooty Shearwater	1, 0	1, 1	1, 0	1, 0	1
	Short-tailed Shearwater	1, 0	1, 1	1, 0	1, 0	1
Seaducks	Black Scoter	6, 0	6, 0	4, 0	6, 0	6
	Harlequin Duck	1, 0	1, 0	0, 0	1, 0	1
	King Eider	3, 1	3, 0	3, 0	3, 0	3
	Spectacled Eider	3, 0	2, 1	1, 0	2, 0	3
	Steller’s Eider	12, 1	12, 0	9, 0	12, 0	12
	White-winged Scoter	5, 0	5, 0	1, 0	5, 0	5
Alcids	Ancient Murrelet	7, 4	5, 3	6, 1	7, 2	7
	Cassin’s Auklet	3, 0	3, 0	1, 0	3, 0	3
	Crested Auklet	8, 2	7, 2	5, 0	8, 0	8
	Kittlitz’s Murrelet	2, 1	2, 1	1, 0	2, 1	2
	Least Auklet	5, 3	3, 1	4, 0	5, 0	5
	Marbled Murrelet	1, 0	1, 0	0, 0	1, 0	1
	Parakeet Auklet	13, 4	11, 4	9, 1	13, 1	13
	Pigeon Guillemot	1, 0	1, 0	0, 0	1, 0	1
	Thick-billed Murre	1, 0	1, 0	1, 0	1, 0	1
	Whiskered Auklet	11, 9	7, 7	11, 1	11, 4	11
Gulls/Terns	Aleutian Tern	1, 0	1, 0	1, 0	1, 0	1
	Black-legged Kittiwake	11, 1	10, 2	7, 0	11, 0	11
	Glaucous Gull	2, 0	2, 0	2, 0	2, 0	2
	Glaucous-winged Gull	25, 6	23, 10	19, 4	24, 4	25
	Red-legged Kittiwake	3, 1	2, 1	3, 0	3, 0	3
	Total	138, 39	120, 38	110, 10	136, 16	138

Areas of highest model agreement for climate vulnerability were throughout the EBS and Central Aleutians (Fig 3). Based on the magnitude-agreement score for shallow SWT, core areas were vulnerable throughout the study area, implicating all 26 species in our study (Table 7). In the Central Aleutians, 16 core areas projected changes greater than 1 SD from baseline conditions, as well as 2 core areas in the Eastern Aleutians. These included 1 core area for Fork-tailed Storm-Petrel (*Oceanodroma furcata)*, 3 for Northern Fulmar (*Fulmarus glacialis)*, 2 for Ancient Murrelet (*Synthliboramphus antiquus)*, 1 for Kittlitz’s Murrelet (*Brachyramphus brevirostris*), 1 for Parakeet Auklet (*Aethia psittacula)*, 4 for Whiskered Auklet (*A*. *pygmaea)*, and 4 for Glaucous-winged Gull (*Larus glaucescens*; Fig 3, Table 7).

#### Deep seawater temperature (SWT)

Like shallow water, deep SWT is projected to increase across most of the study area in all three models. The MIROC model identified 60 of 68 core areas as vulnerable to climate changes (Table 8). Of those, 35 core areas for 11 species within 12 IBAs were in the high vulnerability category across the Western and Central Aleutians (S1 Figs). The CGCM model identified 51 core areas as moderately vulnerable, with 15 rated in the high category for 8 species in 10 IBAs in the Eastern Aleutians and adjacent areas. ECHO-G, which indicated the least warming when averaged across the study area (Tables 3 and 4), indicated the highest number of deep-water core areas as vulnerable, at 65 of 68. Eighteen overlapping core areas for 11 species in 8 IBAs were rated as high vulnerability located primarily in the Western Aleutians.

**Table 8:** Summary of the number of species core areas projected to have moderate (> +0.1 SD change) or high (> +1 SD change) climate vulnerability due to deep seawater temperature increase. Data presented are for each of three climate models analyzed plus the magnitude-agreement (M-A) score summarizing average SD change and agreement across models.

		MIROC	GCGM	ECHO-G	M-A Score	Total Core Areas
Guild	Species	Mod, High	Mod, High	Mod, High	Mod, High	
Petrels	Fork-tailed Storm-Petrel	2, 2	3, 0	4, 1	4, 0	4
	Leach’s Storm-Petrel	1, 1	0, 0	1, 1	1, 0	1
	Northern Fulmar	3, 3	4, 2	4, 1	4, 1	4
	Sooty Shearwater	1, 0	1, 0	1, 0	1, 0	1
	Short-tailed Shearwater	1, 0	1, 0	1, 0	1, 0	1
Seaducks	Harlequin Duck	1, 0	1, 0	1, 0	1, 0	1
	White-winged Scoter	1, 0	1, 0	1, 0	1, 0	1
Alcids	Ancient Murrelet	4, 4	3, 1	5, 2	5, 1	5
	Cassin’s Auklet	1, 0	1, 0	1, 0	1, 0	1
	Crested Auklet	3, 2	2, 1	3, 1	3, 0	3
	Kittlitz’s Murrelet	1, 0	1, 1	1, 0	1, 0	1
	Least Auklet	3, 3	1, 0	3, 2	3, 0	3
	Parakeet Auklet	6, 4	4, 2	6, 2	6, 0	6
	Pigeon Guillemot	0, 0	1, 0	0, 0	0, 0	1
	Whiskered Auklet	11, 9	7, 3	11, 5	11, 1	11
Gulls/Terns	Black-legged Kittiwake	2, 1	2, 1	3, 1	3, 0	3
	Glaucous Gull	0, 0	1, 0	1, 0	1, 0	1
	Glaucous-winged Gull	16, 5	15, 4	15, 1	16, 0	17
	Red-legged Kittiwake	3, 1	2, 0	3, 1	3, 0	3
	Total	60, 35	51, 15	65, 18	66, 3	68

The areas of greatest model agreement were in the southern EBS Outer Shelf, Eastern Aleutians, the Central Aleutians, and most of the GOA (Fig 4). Based on the magnitude-agreement score for deep SWT, core areas were vulnerable throughout the study area, except for two in the Kodiak & Cook Inlet ecoregion (within the Kenai Fjords IBA). Vulnerable core areas included 18 of the 19 species occurring over deep water: all five petrels, both seaducks, seven of eight alcids (all but Pigeon Guillemot (*Cepphus columba*), and all four gulls/terns (Table 8). Three areas of high vulnerability found in the AI were at the Buldir & Near Islands Marine, Kagamil Island Marine, and Chagulak Island Marine IBAs.

#### Ice cover

Concentration of sea ice is projected to decrease throughout the study area in all three models. The MIROC model identified 16 core areas in 13 IBAs as climate vulnerable, with 9 of those core areas for 8 species in 8 IBAs in the high vulnerability category (Table 9) along the EBS Inner and Outer Shelves (S1 Figs). The CGCM model identified 15 core areas as vulnerable, with none rated in the high category. ECHO-G projected 17 core areas as moderate vulnerability as well as 2 as high vulnerability for King Eider on the EBS Inner Shelf.

**Table 9:** Summary of the number of species core areas projected to have moderate (> -0.1 SD change) or high (> -1 SD change) climate vulnerability due to decreasing sea ice concentration. Data presented are for each of three climate models analyzed plus the magnitude-agreement (M-A) score summarizing average SD change and agreement across models.

		MIROC	GCGM	ECHO-G	M-A Score	Total Core Areas
Guild	Species	Mod, High	Mod, High	Mod, High	Mod, High	
Petrels	Fork-tailed Storm-Petrel	1, 1	1, 0	1, 0	1, 0	1
Seaducks	King Eider	2, 1	2, 0	2, 2	2, 0	2
	Spectacled Eider	3, 1	2, 0	3, 0	3, 0	3
	Steller’s Eider	2, 2	2, 0	2, 0	2, 0	2
Alcids	Crested Auklet	3, 0	2, 0	3, 0	3, 0	4
	Least Auklet	2, 1	1, 0	1, 0	1, 0	2
	Parakeet Auklet	1, 1	3, 0	3, 0	3, 0	3
Gulls/Terns	Black-legged Kittiwake	1, 1	1, 0	1, 0	1, 0	1
	Glaucous Gull	1, 1	1, 0	1, 0	1, 0	1
	Total	16, 9	15, 0	17, 2	17, 0	19

Greatest model agreement was found in the southern core areas within the seasonal ice zone (Fig 5). Based on the magnitude-agreement score for sea ice, 17 core areas (leaving out 2 core areas in the Savoonga Colonies IBA) were categorized as climate vulnerable, implicating all 9 species with core areas in the sea ice zone (Table 9).

#### Large copepods

Among the three models, the biomass of large, neritic *Neocalanus* and other similar copepod species was inconsistently projected to increase in some models while decreasing in others for the same cores within the same season (Fig 2). The MIROC model projected the largest increases, with biomass increasing by 0.49 mg C / m^3^ on the EBS Inner Shelf. The model also projected large decreases, up to -0.27 mg C / m^3^ near the Central Aleutians. MIROC identified 23 of 46 core areas as climate vulnerable for 6 of 7 species within 10 IBAs (Table 10) primarily following an arc along the Western and Central and Eastern Aleutians (S1 Figs). Of those, 12 core areas in 6 IBAs were in the high vulnerability category, located in the Western Aleutians. The CGCM model identified two core areas in two IBAs in the EBS Outer Shelf, Eastern Aleutians, and adjacent areas as having moderate vulnerability. Unlike the MIROC model, CGCM projected the greatest decrease in biomass of the three models (-0.31 mg C / m^3^) on the EBS Inner Shelf (Fig 2). ECHO-G identified 13 core areas as vulnerable for 6 species in 7 IBAs scattered throughout the region.

**Table 10:** Summary of the number of species core areas projected to have moderate (> -0.1 SD change) or high (> -1 SD change) climate vulnerability due to decreasing large copepod biomass. Data presented are for each of three climate models analyzed plus the magnitude-agreement (M-A) score summarizing average SD change and agreement across models.

		MIROC	GCGM	ECHO-G	M-A Score	Total Core Areas
Guild	Species	Mod, High	Mod, High	Mod, High	Mod, High	
Petrels	Fork-tailed Storm-Petrel	2, 1	1, 0	2, 0	1, 0	4
	Leach’s Storm-Petrel	1, 1	0, 0	2, 0	1, 0	2
Alcids	Cassin’s Auklet	0, 0	0, 0	1, 0	0, 0	3
	Crested Auklet	4, 1	0, 0	2, 0	1, 0	8
	Least Auklet	4, 2	0, 0	0, 0	0, 0	5
	Parakeet Auklet	4, 3	1, 0	3, 0	1, 0	13
	Whiskered Auklet	8, 4	0, 0	3, 0	1, 0	11
	Total	23, 12	2, 0	13, 0	5, 0	46

There were no core areas where all three models agreed, and the areas with one or two models indicating vulnerability were scattered throughout the study area (Fig 6). Based on the magnitude-agreement score for large copepods, 5 overlapping core areas were climate vulnerable in the Buldir Island Colony in the Western Aleutians.

#### Euphausiids

The MIROC model projected the largest decreases for euphausiid biomass, declining by up to -1.85 mg C / m^3^ in the Central Aleutians. MIROC identified as climate vulnerable 39 of 107 core areas within 18 IBAs throughout the AI as well as the Eastern Aleutians and adjacent areas, implicating 12 of 19 species (S1 Figs, Table 11). Of those, six IBAs in the AI were in the high vulnerability category. The CGCM model identified 30 core areas for 12 species in 21 IBAs as moderate vulnerability, with 3 species in 9 IBAs in the high category, most numerous on the EBS Outer Shelf, Eastern Aleutians, and Alaska Peninsula South. Contrary to the MIROC model, CGCM projected the greatest increase in biomass of the three models (up to +1.26 mg C / m^3^) in the Western Aleutians. ECHO-G identified 43 moderately vulnerable core areas for 18 species in 21 IBAs primarily in the EBS and AI, of which 2 were highly vulnerable in the Central Aleutians. Core areas were identified as vulnerable by all three models for Fork-tailed Storm-Petrel, Northern Fulmar, Ancient Murrelet, Crested Auklet (*A*. *cristatella)*, Parakeet Auklet, Whiskered Auklet, Black-legged Kittiwake, Glaucous Gull, Glaucous-winged Gull, and Red-legged Kittiwake (Table 11).

**Table 11:** Summary of the number of species core areas projected to have moderate (> -0.1 SD change) or high (> -1 SD change) climate vulnerability due to decreasing euphausiid biomass. Data presented are for each of three climate models analyzed plus the magnitude-agreement (M-A) score summarizing average SD change and agreement across models.

		MIROC	GCGM	ECHO-G	M-A Score	Total Core Areas
Guild	Species	Mod, High	Mod, High	Mod, High	Mod, High	
Petrels	Fork-tailed Storm-Petrel	2, 0	1, 0	2, 0	0, 0	4
	Leach’s Storm-Petrel	1, 0	0, 0	1, 0	0, 0	2
	Northern Fulmar	1, 1	3, 0	4, 1	2, 1	6
	Sooty Shearwater	0, 0	0, 0	1, 0	0, 0	1
	Short-tailed Shearwater	0, 0	0, 0	1, 0	0, 0	1
Alcids	Ancient Murrelet	3, 0	1, 0	3, 0	0, 0	7
	Cassin’s Auklet	0, 0	1, 0	1, 0	0, 0	3
	Crested Auklet	3, 0	2, 0	3, 0	1, 0	8
	Kittlitz’s Murrelet	0, 0	0, 0	1, 0	0, 0	2
	Least Auklet	3, 1	0, 0	1, 0	0, 0	5
	Marbled Murrelet	0, 0	0, 0	0, 0	0, 0	1
	Parakeet Auklet	4, 0	1, 0	4, 0	0, 0	13
	Thick-billed Murre	0, 0	1, 0	1, 0	0, 0	1
	Whiskered Auklet	8, 1	1, 0	4, 0	1, 0	11
Gulls/Terns	Aleutian Tern	0, 0	0, 0	1, 0	0, 0	1
	Black-legged Kittiwake	1, 0	3, 0	3, 0	0, 0	11
	Glaucous Gull	1, 0	2, 1	2, 0	1, 0	2
	Glaucous-winged Gull	11, 3	13, 7	9, 1	8, 1	25
	Red-legged Kittiwake	1, 0	1, 1	1, 0	1, 0	3
	Total	39, 6	30, 9	43, 2	14, 2	107

Areas of greatest model agreement were in the EBS Outer Shelf and Eastern Aleutians (Fig 7). Based on the magnitude-agreement score for euphausiids (Fig 7), 14 areas of moderate vulnerability for 12 IBAs show up primarily in the EBS and the Central Aleutians with 2 areas of high vulnerability in the Chagulak Island Marine and Fenimore Pass & Atka Island Marine IBAs in the Central Aleutians.

#### Benthic infauna

Like large copepods and euphausiids, across the three models, the biomass of benthic infaunal biomass was inconsistently projected to increase in some locations while reportedly decreasing in those same areas by other models. MIROC identified 8 of 31 core areas as moderate vulnerability within 7 IBAs in the Kodiak & Cook Inlet region (S1 Figs, Table 12). The CGCM model was the most extreme of the models, identifying 27 climate-vulnerable core areas in 17 IBAs along the EBS Inner Shelf, Kodiak & Cook Inlet, and one area on the NBS Middle Shelf. The CGCM projected the greatest decreases in biomass of the three models (-0.31 mg C / m^2^), occurring on the EBS Inner and Outer Shelves; to the contrary, the MIROC model indicated increased benthic infaunal biomass across much of the same area. ECHO-G identified only three moderately vulnerable core areas, also on EBS Inner Shelf.

**Table 12:** Summary of the number of species core areas projected to have moderate (> -0.1 SD change) or high (> -1 SD change) climate vulnerability due to decreasing biomass of benthic infauna. Data presented are for each of three climate models analyzed plus the magnitude-agreement (M-A) score summarizing average SD change and agreement across models.

		MIROC	GCGM	ECHO-G	M-A Score	Total Core Areas
Guild	Species	Mod, High	Mod, High	Mod, High	Mod, High	
Seaducks	Black Scoter	2, 0	5, 0	0, 0	1, 0	6
	Harlequin Duck	0, 0	1, 0	0, 0	0, 0	1
	King Eider	0, 0	3, 0	2, 0	1, 0	3
	Spectacled Eider	0, 0	1, 0	0, 0	0, 0	3
	Steller’s Eider	4, 0	12, 0	1, 0	2, 0	12
	White-winged Scoter	2, 0	4, 0	0, 0	0, 0	5
Alcids	Pigeon Guillemot	0, 0	1, 0	0, 0	0, 0	1
	Total	8, 0	27, 0	3, 0	4, 0	31

There were no core areas where all three models agreed; all but two core areas were identified as vulnerable by at least one model (Fig 8). Based on the magnitude-agreement score for benthic infauna, only four core areas were identified as climate vulnerable on the EBS Inner Shelf (Nushagak & Kvichak and Izembek Lagoon & Bechevin Bay IBAs) and at Kodiak & Cook Inlet region (Marmot Bay and Sitkinak Strait IBAs) for King Eider (*Somateria spectabilis*), Steller’s Eider (*Polysticta stelleri*), and Black Scoter (*Melanitta americana*) (Fig 8, Table 12).

### Cross-variable summary

**Fig 9:**
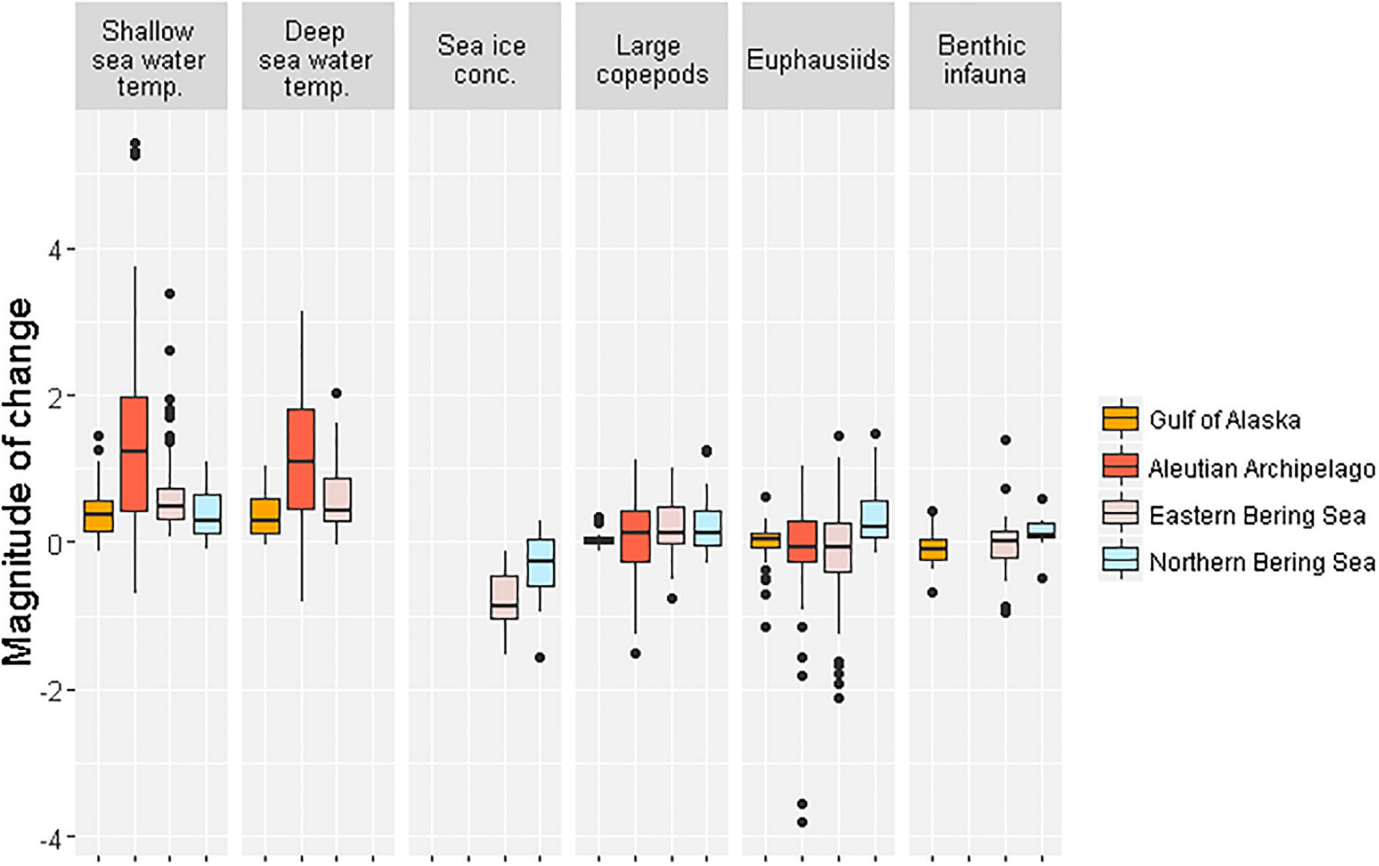
Distribution of standard deviation change values (magnitude of change) for all three models across marine bird core areas grouped by Large Marine Ecosystems.

The distribution of SD change values across all core areas and models varied among LME groups, but were on average most extreme for SWT and sea ice variables (Fig 9, S2 Table). Forage variables varied by projecting both increases and decreases with some extreme negative outliers, for example for euphausiids.

The GOA projections indicated warming SWT and generally increasing copepods. Euphausiid changes varied with several negative outliers; some models showed a decrease in benthic forage resources for several core areas in the Kodiak & Cook Inlet ecoregion. In part due to the narrow range of variability (Table 4), the AI core areas indicated the greatest vulnerability to shallow and deep SWT warming, with median SD change values more than double that of any other LME and some extremely high shallow SWT increases clustered in the eastern portion of the Central Aleutians ecoregion. Values varied for LTLs, showing both increases and decreases, but also with some extremely high euphausiid biomass losses across the AI and notable copepod decreases around Buldir Island.

The EBS core areas had the second-highest median scores for SWT warming with some notably high shallow SWT increases near Unimak Pass and Kuskokwim Bay, with the greatest sea ice loss across core areas (Fig 9). Large copepods were largely projected to increase in biomass, although the Unimak & Akutan Passes IBA stands out as a negative outlier. Euphausiids and benthic infauna varied but with a tendency toward lower biomass. Euphausiid biomass losses of significance were projected by one or more models for 15 different IBAs across the LME. Benthic biomass decreases were notable for areas along the EBS Inner Shelf. The models indicated SWT warming in the NBS core areas, and pronounced sea ice losses relative to their baseline conditions. Forage variables tended to indicate increasing biomass in this LME, but with a few notable decreases indicated by one or more models for the area surrounding St. Lawrence Island.

The combined model results presented much greater vulnerability agreement for projected physical changes than for biological changes (Table 13). For biological forage variables, the three models projected variable climate vulnerability, resulting in lower model agreement, and accordingly, lower magnitude-agreement scores (Table 13; S3 Table).

**Table 13:** Scores for percent of climate-vulnerable core areas, vulnerability agreement among models, projected magnitude of change, and the magnitude-agreement score for each of six physical and biological variables, for marine bird core areas grouped by Large Marine Ecosystems (LMEs).

Ecoregion	Metric	Shallow SWT	Deep SWT	Sea Ice	Large Copepods	Euphausiids	Benthic Infauna
Gulf of Alaska	# core areas tested	39	13	na	8	28	11
	% vulnerable ^a^	100%	100%	na	25%	50%	100%
	vulnerability agreement ^b^	78%	82%	na	8%	20%	48%
	Magnitude ^c^	+0.40	+0.41	na	+0.04	<+0.01	-0.08
	magnitude-agreement score ^d^	+0.31	+0.34	na	<+0.01	<+0.01	-0.04
Aleutian Islands	# core areas tested	35	35	na	21	35	na
	% vulnerable	100%	100%	na	95%	91%	na
	vulnerability agreement	82%	84%	na	40%	42%	na
	magnitude	+1.36	+1.04	na	-0.04	-0.26	na
	magnitude-agreement score	+1.12	+1.04	na	-0.04	-0.26	na
Eastern Bering Sea	# core areas tested	52	20	9	9	35	17
	% vulnerable	100%	100%	100%	67%	89%	100%
	vulnerability agreement	99%	93%	100%	22%	48%	41%
	magnitude	+0.61	+0.58	-0.80	+0.18	-0.14	-0.03
	magnitude-agreement score	+0.60	+0.54	-0.80	+0.04	-0.07	-0.01
Northern Bering Sea	# core areas tested	12	na	10	8	9	3
	% vulnerable	100%	na	100%	63%	11%	33%
	vulnerability agreement	78%	na	70%	21%	3%	11%
	magnitude	+0.37	na	-0.33	+0.27	+0.33	+0.11
	magnitude-agreement score	+0.29	na	-0.23	+0.06	+0.01	+0.01

^a^ % vulnerable = the number of core areas tested that were climate vulnerable based on one or more models.

^b^ vulnerability agreement = number of models (out of a possible three) indicating climate vulnerability, summed across all core areas, divided by the total possible agreement score (the number of core areas times three).

^c^ magnitude = the average of SD change values across all core areas.

^d^ magnitude-agreement score = the magnitude multiplied by the vulnerability agreement, which indicates the relative amount of projected change mediated by the relative agreement among models.

Vulnerability agreement for SWT ranged from 78% to 99%, with highest agreement for the EBS and significant warming for core areas across all LMEs (Table 13). Relative to baseline values, the AI core areas are projected to see the most SWT warming with an extreme average magnitude-agreement score of greater than 1 SD. There was 100% vulnerability agreement for sea ice in the EBS, and both the EBS and NBS core areas projected a significant oncoming decrease in sea ice.

However, vulnerability agreement was lower for biological variables, ranging from 3% to 48%. There was low agreement for large copepods in the GOA core areas (8%), but moderate agreement in the other three LMEs (21–40%). For euphausiids, there was moderate agreement for all LMEs (20–48%) but the NBS (3%). Based on this assessment, LTLs appear to be most vulnerable in the AI; euphausiids have the most significant magnitude-agreement scores in the AI (-0.26) and EBS (-0.14). Notably, all core areas for benthic-feeding birds in the GOA and EBS were found vulnerable by at least one model, although agreement was lower (41–48%) and the magnitude-agreement score lower still (-0.01– -0.04).

All three models agreed that 86 of 138 core areas were vulnerable for shallow SWT warming, 42 of 68 for deep SWT warming, 12 of 19 for sea ice decline, and 6 of 107 for euphuasiids biomass decline; no areas for large copepods or benthic infauna had complete model agreement (Figs 3–8, S3 Table). All 26 species studied were identified as climate vulnerable for one or more variables (Table 14). Although not all of the models projected SWT vulnerability for every core area, when applying the magnitude-agreement score to analyze three-model average change and model agreement, virtually every core area was flagged as climate vulnerable for SWT (both shallow and deep) and sea ice changes(136/138, 66/68, and 17/19 respectively; Table 14). Based on the magnitude-agreement score, about half of the marine bird species were climate vulnerable for one or more forage variables (Table 14 and S4 Table).

**Table 14:** Number of climate-vulnerable core areas out of total number assessed, based on the magnitude-agreement score. Species in bold have at least one climate-vulnerable core area for forage resources. The last column indicates the number of core areas vulnerable in all categories assessed.

Guild	Species	Shallow SWT	Deep SWT	Sea ice	Large copepods	Euphausiids	Benthic infauna	All Variables
Petrels	**Fork-tailed Storm-Petrel**	4/4	4/4	1/1	1/4	0/4		
	**Leach’s Storm-Petrel**	2/2	1/1		1/2	0/2		
	**Northern Fulmar**	6/6	4/4			2/6		2/6
	Sooty Shearwater	1/1	1/1			0/1		
	Short-tailed Shearwater	1/1	1/1			0/1		
Seaducks	**Black Scoter**	6/6					1/6	1/6
	Harlequin Duck	1/1	1/1				0/1	
	**King Eider**	3/3		2/2			1/3	1/3
	Spectacled Eider	2/3		3/3			0/3	
	**Steller’s Eider**	12/12		2/2			2/12	2/12
	White-winged Scoter	5/5	1/1				0/5	
Alcids	Ancient Murrelet	7/7	5/5			0/7		
	Cassin’s Auklet	3/3	1/1		0/3	0/3		
	**Crested Auklet**	8/8	3/3	3/4	1/8	1/8		
	Kittlitz’s Murrelet	2/2	1/1			0/2		
	Least Auklet	5/5	3/3	1/2	0/5	0/5		
	Marbled Murrelet	1/1				0/1		
	**Parakeet Auklet**	13/13	6/6	3/3	1/13	0/13		
	Pigeon Guillemot	1/1	0/1				0/1	
	Thick-billed Murre	1/1				0/1		
	**Whiskered Auklet**	11/11	11/11		1/11	1/11		
Gulls/Terns	Aleutian Tern	1/1				0/1		
	Black-legged Kittiwake	11/11	3/3	1/1		0/11		
	**Glaucous Gull**	2/2	1/1	1/1		1/2		1/1
	**Glaucous-winged Gull**	24/25	16/17			8/25		8/25
	**Red-legged Kittiwake**	3/3	3/3			1/3		1/3
	Total	136/138	66/68	17/19	5/46	14/107	4/31	16/138

**Fig 10:**
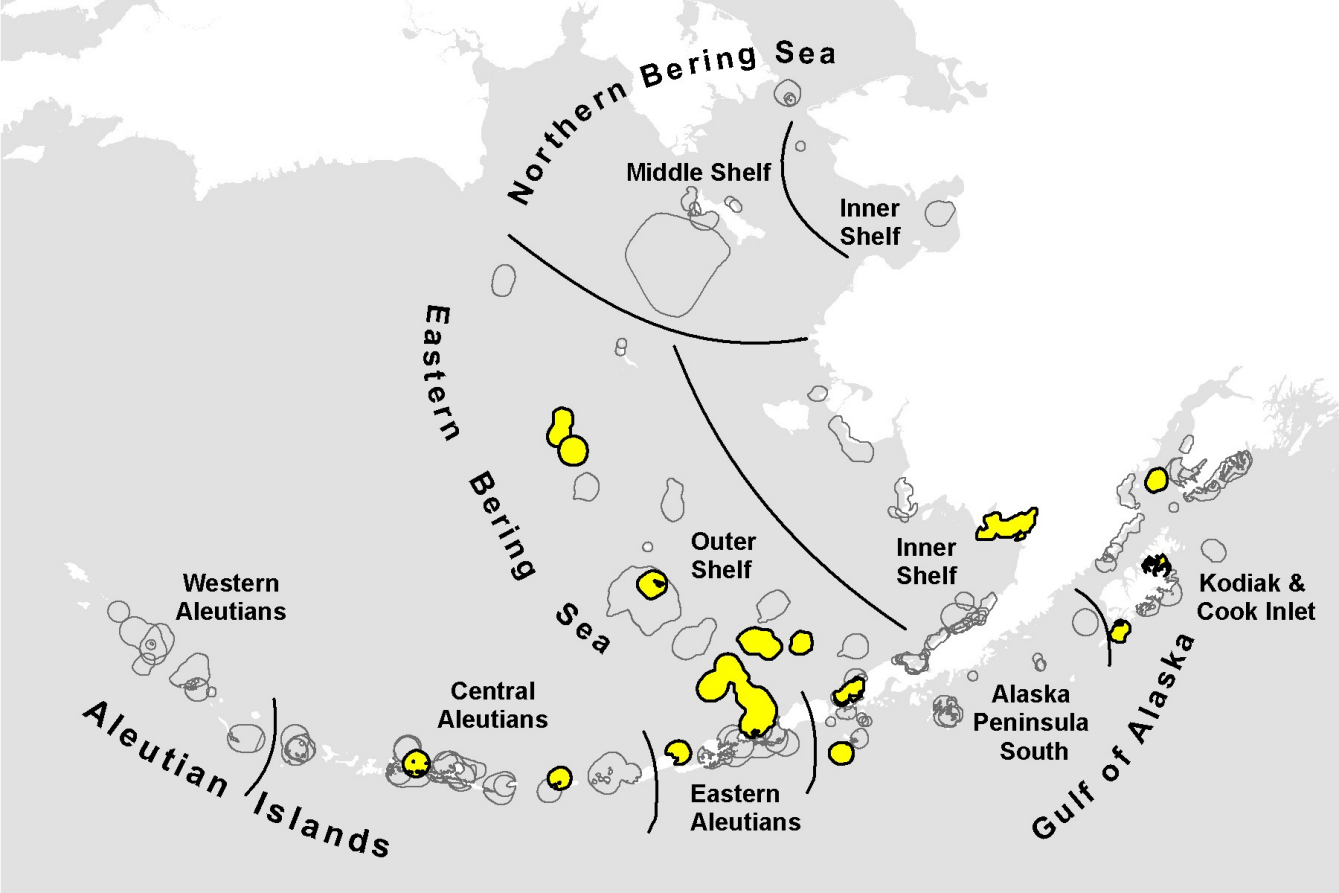
Core areas that are climate vulnerable for all physical and biological variables assessed, based on the magnitude-agreement score.

Overall, 12% of core areas were climate vulnerable for all variables assessed, including 16 core areas within 14 IBAs (Fig 10). This included 1 core area in the Alaska Peninsula South for Glaucous-winged Gull; 3 in Kodiak & Cook Inlet for Black Scoter, Steller’s Eider, and Glaucous-winged Gull; 2 in the Central Aleutians for Glaucous-winged Gull and Northern Fulmar; 2 in the Eastern Aleutians for Glaucous-winged Gull; 1 in the EBS Inner Shelf for King Eider; and 5 in the EBS Outer Shelf for Northern Fulmar, Glaucous and Glaucous-winged Gulls, and Red-legged Kittiwake (Fig 10, Table 14).

## Discussion

Hermann et al. [18] recognized a broad rise in SWT based on this suite of models, exceeding 1.5° C in some areas, with a corresponding loss in sea ice cover. Likewise in our study, at the project area scale, physical changes were significant for all models for all seasons, except for the ECHO-G model in winter (Table 4). The adverse physical effects carried over to the other scales of analysis as well, with all LMEs (Table 6) and nearly every marine bird core area flagged as climate vulnerable for substantial SWT and sea ice changes (Figs 3–8). If the models are accurate, these physical changes will reach every marine bird species (Table 14) and IBA (S4 Table) included in our study.

### Variability and agreement

About two-thirds of core areas had full model vulnerability agreement for physical variables, resulting in 89–99% of core areas classified as vulnerable under the magnitude-agreement score (S3 and S4 Tables). Results were less convergent for the biological variables, which varied across models and regions, resulting in 11–13% of core areas marked as vulnerable using the magnitude-agreement score (S3 and S4 Tables). While the majority of core areas were identified as climate vulnerable for forage variables by one or more models (72% for large copepods, 73% for euphausiids, and 94% for benthic infauna), very few were agreed upon by all three models (only 6% of euphausiid-forager core areas). LTL biomass declines were most significant in the winter season (Table 4), and greatest agreement was found for euphausiids, where ~90% of core areas in the AI and EBS were vulnerable by one or more models, and 6 areas were flagged by all three models (Fig 7). For benthic biomass in the GOA and EBS, all core areas were identified as climate vulnerable by one or more models (Table 13), but none by all three models (Fig 8). For large copepods in the AI, 95% were flagged by one or more models, but none by all three (Fig 6). Low agreement among models points to the high complexity in parameterization of individual models to project future system dynamics.

These models make up a small ensemble from which to interpret the breadth of potential futures; in response, we integrated model agreement as well as magnitude of change into overall climate vulnerability scores to identify and highlight areas with the greatest certainty of projected impacts. We often found that MIROC, GCGM, and ECHO-G confoundingly disagreed on the direction of change for forage variable biomass within the same localized region, resulting in low magnitude-agreement scores and fewer core areas identified as climate vulnerable by our study. For example, benthic biomass values exhibited the importance of assessing multiple model realizations. Applying CGCM only, core areas in the EBS Inner Shelf appeared vulnerable to loss of forage biomass; yet, when combined with the MIROC and ECHO-G model realizations, model agreement waned. This speaks to the importance of using multiple models to assess changes, and cautiously interpreting results where model projections are not aligned.

In the finest scale of our assessment, we analyzed marine bird core areas and described those results grouped by ecoregion and LME to look for regional patterns. Modeled systems covering a broad geography are limited in their ability to reproduce high-resolution field measurements for a precise spatial location and time; yet models should align with field studies in their general direction, magnitude, and rate of change. Interpretation of the forage variables from these models was complicated due to the often lack of agreement on the direction and magnitude of change, and the resulting lack of climate-vulnerable core areas identified.

The results are further influenced by the normal range of variability of the reference LMEs. For example, the seasonally ice-covered EBS and NBS have lower averages and greater variability in SWT over the year than the AI and GOA where smaller shifts in temperature can result in conditions outside of the normal range. Similarly, although the EBS is projected to lose more ice (Table 5), the NBS has a greater annual average projected magnitude of change (Table 6). The timing of sea ice retreat, rather than the extent or concentration, may be the most important sea ice factor affecting seabird distribution [14, 15].

In alignment with the results of our study which generally indicated greater shifts for marine bird core areas in the AI, EBS, and GOA than the NBS (Fig 9), Alabia et al. [63] found that sub-Arctic species of marine invertebrates and fishes were more sensitive to climate changes than Arctic species and concluded that species-specific vulnerability assessments were necessary to account for differences in responses to observed climate shifts. Nonetheless, an oncoming major shift in the benthic-pelagic coupling of the NBS ecosystem is predicted [64], and we recognize that at fine scales a multitude of factors, physical and biological, could alter or magnify the effects of environmental drivers on LTLs and benthic infauna. For example, documented changes in hydrography and export production, as well as northward movement of pelagic predators into the NBS, have potential to influence LTL distribution and abundance [20, 65]. In the Bering and Chukchi Seas, predicted increases in zooplankton grazers and upper trophic predators could lead to decreases in sedimentation and ultimately lower benthic biomass and altered community composition [65]. Increases in large whales in the NBS region may have already impacted zooplankton and benthic infauna [66, 67].

In this study, we calculated temporal averages to assess conditions seasonally, but on a decadal timescale. Our analysis smooths out interannual variability and some of the finest scale heterogeneity in resource patterns, making it more difficult to detect acute changes and contributing to the models yielding less clear trends than those from field studies (e.g., [28]). Analysis over longer time periods may obscure shorter periods of time where critical ecological thresholds are exceeded. For example, changes in forage quality during pre-breeding months have carry-over effects in terms of seabird reproductive success even if forage quality improves over the remaining months [68]. Likewise, adult survival can be affected by winter ocean conditions, with impacts on population trends, as identified in models based on well-studied populations of Cassin’s Auklet in the California Current [69]. Such ecological signals that are critical to specific life history phases would be missed using a full-year or multi-year average. Additionally, because our results are averaged over longer periods of time, abrupt extreme events have been smoothed out. We recommend analyzing the same climate models used in this study to identify the projected frequency of extreme events to supplement our study focused on the mean change over time. Such a study could seek to identify the ocean conditions leading to recent seabird mass mortality events in the North Pacific [30–32] and look for similar signals in projected data.

### Most vulnerable IBAs and species

The Central and Eastern Aleutians were highly vulnerable to shallow SWT changes, with magnitude-agreement scores greater than 1 SD. Most notably, the Fenimore Pass & Atka Island Marine IBA included 7 of these 16 highly vulnerable core areas, affecting Ancient Murrelet, Parakeet Auklet, Whiskered Auklet, and Glaucous-winged Gull. Other species with highly vulnerable core areas were Fork-tailed Storm-Petrel, Northern Fulmar, and Kittlitz’s Murrelet. Two of the same core areas in the Central Aleutians for Northern Fulmar and Whiskered Auklet were highly vulnerable for deep SWT as well, at Chagulak Island Marine and Kagamil Island Marine IBAs. Chagulak Island Marine and Fenimore Pass & Atka Island Marine IBAs were also found highly vulnerable to euphausiid decline for Northern Fulmar and Glaucous-winged Gull. These areas of high vulnerability and high model agreement constitute the greatest concern resulting from our study.

The Unimak & Akutan Passes IBA included the highest number of climate-vulnerable core areas (eight for SWT and two of those also for euphausiids). Notably, this IBA supports more than 60 species, has globally significant populations of 14 species, and has the highest bird abundance of any IBA in Alaska, estimated around 7 million, primarily shearwaters [51, 70]. It was also identified as having the highest shipping accident risk to marine birds in the Aleutians [71, 72], which compounds the potential for cumulative effects.

Tied with Unimak & Akutan Passes was the Buldir Island Colony IBA in the Western Aleutians with eight vulnerable core areas (all vulnerable for SWT and five of those also for large copepods). At Buldir Island some repeating species—Whiskered Auklet, Fork-tailed Storm-Petrel, and Parakeet Auklet—were found moderately vulnerable for large copepod biomass decline, along with core areas for Leach’s Storm-Petrels and Crested Auklet. This colony is the largest within Alaska with 3.6 million nesting birds of 20 species, and according to a recent study is among the top-ranked ecological units in the Bering Sea for bird ecological value [72, 73]. This area is vulnerable to the effects of commercial fishing and vessel traffic which both occur in relatively high density near the island [71, 72].

Euphausiid vulnerability was clustered in the Eastern Aleutians and the EBS Outer Shelf, implicating several pelagic Bering shelf and shelf edge IBAs, as well as St. George Island Marine, Izembek Lagoon & Bechevin Bay, Cape Tanak Marine, Gulf of Alaska Shelf Edge, and Lower Cook Inlet IBAs. Again, these projected changes would affect Northern Fulmar, Glaucous-winged Gull, Crested Auklet, and Whiskered Auklet, with the addition of Glaucous Gull and Red-legged Kittiwake. One-third of Glaucous-winged Gull core areas were vulnerable to significantly declining euphausiid biomass.

Another recent study of climate effects on seabirds for this region found that kittiwakes were vulnerable to significant increases in SWT and decreases in LTL productivity, and that warming in recent decades affected the predictability of the available prey base [74]. None of these species rely solely on euphausiids, but the forage fish they also consume depend on euphausiids as well. Based on known diets, those most likely to be impacted would be the auklets (which also consume copepods and larval fish) [75]. The high vulnerability findings for the Aleutians could have important implications to the Whiskered Auklet, which is endemic to the region, and where most of the world population breeds and overwinters [76]. Indeed, euphausiids support large numbers of breeding and migrating birds throughout the Aleutians in summer, and even into the winter in the Unimak & Akutan Passes IBA [76].

Substantial benthic infaunal declines were projected along the EBS Inner Shelf and the Gulf of Alaska for Steller’s Eider, King Eider, and Black Scoter. Notably, this included two sensitive areas, Izembek Lagoon & Bechevin Bay and Nushagak & Kvichak Bays IBAs. Izembek Lagoon is important to migrating, molting, and winter Steller’s Eiders which are a threatened species under the US Endangered Species Act [77, 78]. Steller’s Eider annual survival estimates have been linked to climate conditions, indicating that warmer periods have a negative influence on survival while cooler conditions enhanced survival [79]. Izembek is also a highly important staging area for the >90% of the Pacific population of Black Brant (not included in this study) that stop over at Izembek in the greatest abundance of 11 major staging sites from Mexico to Alaska [80, 81]. Brant appear to be increasing their winter use of Izembek, aided by warmer years allowing ice-free access to eelgrass beds [82]. Although designated wilderness, this national wildlife refuge is the subject of potential development from a proposed road which has raised concerns about habitat security for these and other species that rely on the lagoon [81]. Nushagak and Kvichak Bays, located in inner Bristol Bay, may be subject to development of one of the world’s largest copper and gold mines near their headwaters, which has raised concerns about contamination from toxic tailings [83]. A variety of marine birds rely on Bristol Bay during their annual life cycle, and Steller’s and King Eiders could face deleterious industrial and climate effects in coming decades [84].

Spatially clustered individual-model vulnerability scores make St. Lawrence Island another area of concern. Spectacled Eiders in the St. Lawrence Island Polynya IBA were vulnerable to sea ice loss based on the magnitude-agreement score, and the analysis also indicated vulnerability for benthic infauna based on the GCGM model. The St. Lawrence Island Polynya IBA is the wintering grounds of the global population of Spectacled Eiders, estimated at around 350,000 individuals [85], and may be critical to overwintering survival and preparation for spring movements to breeding areas [86]. Changes in the species composition of key prey in this IBA, from larger to smaller bivalve species, already has implications for the eider’s ability to survive and prepare for breeding [87]. Crested and Least Auklets at the Savoonga Colonies IBA were vulnerable for copepod biomass decreases based on the MIROC model; and Crested Auklets in the Western St. Lawrence Island Marine IBA were vulnerable for euphausiid biomass decrease based on the ECHO-G model. In summer, St. Lawrence Island supports millions of breeding auklets of both species, which forage in the Anadyr Current that advects a high biomass of copepods and euphausiids through the region, a resource critical to successful chick-rearing [50, 88, 89].

Most species in our study forage on fish as a major component of their diet (S1 Table); without the ability to assess climate vulnerability for the complete diet, there are too many unknowns to draw strong conclusions about these species’ future. Indirectly, however, marine birds could be affected by changes in forage fish resulting from the changes identified in our models, both physical and biological. Forage fish could be impacted by access to adequate zooplankton for food, and additionally, the extent of the deep water cold pool of the Bering Sea affects fish distribution, and as it shrinks, large predatory fish move in from the south [20]. Negative effects on survival of juvenile Walleye Pollock (*Theragra chalcogramma*), a key prey of piscivorous seabirds in the Bering Sea, are predicted due to warming seas affecting zooplankton and increased predation on juvenile Pollock [90].

### Conclusion

Overall, our study indicates that no species we assessed is without some level of climate vulnerability. The climate-vulnerable IBAs and species identified here warrant further consideration, which may include assessment of other compounding stressors like intensified vessel traffic or other industrial activities, or complications related to ocean acidification. These areas could also benefit from additional climate modeling studies (especially those predictive of forage fish density), retrospective analyses relating population trends to forage availability (e.g., [91]), increased monitoring, and/or increased conservation measures.

Ultimately, seabirds will need to follow the food, and with climate change, species’ ranges may shrink, expand, or shift and there will be winners and losers [92]. An example of winners is the group of three North Pacific albatross species, which have increased in the Aleutians and Bering Sea, and which may be partly due to increased squid biomass [93]. Farther north in the Chukchi Sea, planktivorous seabirds have recently become predominate in offshore waters, where piscivorous birds once prevailed [94]. However, the planktivorous species (primarily *Aethia* auklets and migratory shearwaters) do not breed along the Chukchi coast, and based on our results, auklet populations could eventually be impacted by breeding conditions in the Bering Sea, regardless of conditions in the Chukchi Sea. Avian response to climate changes might include adaptation in place or emigration to new areas as habitat suitability shifts. Climate modeling studies such as ours can identify areas where novel foraging hotspots, for example, might appear. Future studies could incorporate these data as well as bird distribution data to identify emerging places that may become important to birds as well as places that may no longer support current bird populations. Additionally, these data could be applied to design field studies that measure LTLs and benthic biomass over time to test the hypothesis that forage biomass will increase, and those results could be employed to improve climate models.

The strong signal of changing physical conditions underscores the need for cautious and conservative management (e.g., establishing or maintaining protected areas [87, 95] or best management practices). Temperature increases like those projected by these models can negatively affect seabirds as well as forage prey. For example, warming sea temperature reduces the lipid-rich zooplankton important to many upper trophic level foragers [28]. Recent mass mortality events involving multiple species of planktivorous and piscivorous seabirds and marine mammals suggest a real-world signal that climate warming has and will negatively impact upper trophic foragers. In the highly studied California Current, projected increases in SWT were identified as driving forces in predicted population declines for the planktivorous Cassin’s Auklet as well as decline in habitat suitability for piscivorous Tufted Puffins, likely due to impacts to zooplankton and forage fish timing, duration, and abundance [69, 96]. Ultimately, modeling future biological responses to changing physical drivers is very complex, with incomplete knowledge about the future variability in forage resources introducing substantial variability among model realizations. Even with uncertainty about future biomass and availability of prey species, the high level of climate vulnerability for SWT and sea ice changes indicated climate vulnerability throughout the study area.

## Supporting information

S1 Table Main forage types of marine bird species included in this assessment. Entries with ×× indicate most common prey items.
(PDF)

S2 Table Three-model average magnitude of change, by species core area. Values in bold exceed the climate vulnerability threshold, prior to integrating the model vulnerability agreement score.
(PDF)

S3 Table Model vulnerability agreement, by species core area. Values in bold indicate core areas where all three model results exceeded the climate vulnerability threshold.
(PDF)

S4 Table Three-model average magnitude-agreement scores, by species core area. (PDF)

S1 Figs Climate vulnerability maps by variable and model. (PDF)
