# Supplementary material for: An assessment of climate change vulnerability for Important Bird Areas in the Bering Sea and Aleutian Arc
Source: PLoS One. 2019 Apr 17;14(4):e0214573. doi: 10.1371/journal.pone.0214573 (PMC6469780; doi:10.1371/journal.pone.0214573)
Supplement: S1 Table — Entries with ×× indicate most common prey items. (PDF) [file pone.0214573.s001.pdf]

S1 Table. Main forage types of marine bird species included in this assessment.

Guild	Species	Scientific Name	Large copepods	Euphausiids	Benthic infauna	Fish & cephalopods
Petrels	Fork-tailed Storm-Petrel	*Oceanodroma furcata*	×	×		×
	Leach's Storm-Petrel	*Oceanodroma leucorhoa*	×	×		×
	Northern Fulmar	*Fulmarus glacialis*		×		×
	Sooty Shearwater	*Ardenna griseus*		×		×
	Short-tailed Shearwater	*Ardenna tenuirostris*		×		×
Seaducks	Black Scoter	*Melanitta americana*			×	
	Harlequin Duck	*Histrionicus histrionicus*			×	
	King Eider	*Somateria spectabilis*			×	
	Spectacled Eider	*Somateria fischeri*			×	
	Steller's Eider	*Polysticta stelleri*			×	
	White-winged Scoter	*Melanitta deglandi*			×	
Alcids	Ancient Murrelet	*Synthliboramphus antiquus*		×		×
	Cassin's Auklet	*Ptychoramphus aleuticus*	×	×		
	Crested Auklet	*Aethia cristatella*	×	×		
	Kittlitz's Murrelet	*Brachyramphus brevirostris*		×		×
	Least Auklet	*Aethia pusilla*	×	×		
	Marbled Murrelet	*Brachyramphus marmoratus*		×		×
	Parakeet Auklet	*Aethia psittacula*	×	×		×
	Pigeon Guillemot	*Cepphus columba*			×	×
	Thick-billed Murre	*Uria lomvia*		×		×
	Whiskered Auklet	*Aethia pygmaea*	×	×		
Gulls/Terns	Aleutian Tern	*Onychoprion aleuticus*		×		×
	Black-legged Kittiwake	*Rissa tridactyla*		×		×
	Glaucous Gull	*Larus hyperboreus*		×		×
	Glaucous-winged Gull	*Larus glaucescens*		×		×
	Red-legged Kittiwake	*Rissa brevirostris*		×		×
