# Supplementary material for: An assessment of climate change vulnerability for Important Bird Areas in the Bering Sea and Aleutian Arc
Source: PLoS One. 2019 Apr 17;14(4):e0214573. doi: 10.1371/journal.pone.0214573 (PMC6469780; doi:10.1371/journal.pone.0214573)
Supplement: S2 Table — Values in bold exceed the climate vulnerability threshold, prior to integrating the model vulnerability agreement score. (PDF) [file pone.0214573.s002.pdf]

S2 Table. Three-model average magnitude of change, by species core area. Values in bold exceed the climate vulnerability threshold, prior to integrating the model vulnerability agreement score.

LME	Species	IBA	Season	Shallow SWT	Deep SWT	Sea Ice Cover	Large Copepods	Euphausiids	Benthic Infauna
Aleutian Islands	ANMU	Buldir & Near Islands Marine	Summer	**1.004**	**1.591**			0.150	
Aleutian Islands	ANMU	Buldir Island Colony	Summer	**0.583**	**1.171**			-0.022	
Aleutian Islands	ANMU	Fenimore Pass & Atka Island Marine	Summer	**2.237**	**0.879**			0.134	
Aleutian Islands	ANMU	Koniuji-Atka Island Colony	Summer	**2.054**	**0.867**			0.104	
Aleutian Islands	BLKI	Buldir Island Colony	Summer	**0.583**	**1.171**			-0.022	
Aleutian Islands	CRAU	Buldir Island Colony	Summer	**0.583**	**1.171**		**-0.411**	-0.022	
Aleutian Islands	CRAU	Gareloi Island Marine	Summer	**1.074**	**0.887**		0.197	-0.007	
Aleutian Islands	FTSP	Buldir Island Colony	Summer	**0.583**	**1.171**		**-0.411**	-0.022	
Aleutian Islands	FTSP	Koniuji-Atka Island Colony	Summer	**2.054**	**0.867**		0.159	0.104	
Aleutian Islands	GWGU	Buldir & Near Islands Marine	Summer	**0.165**	**0.625**			0.024	
Aleutian Islands	GWGU	Fenimore Pass & Atka Island Marine	Summer	**2.239**	**0.892**			0.136	
Aleutian Islands	GWGU	Fenimore Pass & Atka Island Marine	Summer	**1.935**	**0.926**			-0.080	
Aleutian Islands	GWGU	Fenimore Pass & Atka Island Marine	Winter	**2.142**	**0.929**			**-0.842**	
Aleutian Islands	GWGU	Kiska Island Colonies	Summer	**0.409**	**0.584**			0.053	
Aleutian Islands	LEAU	Gareloi Island Marine	Summer	**1.066**	**0.883**		0.197	-0.007	
Aleutian Islands	LEAU	Kiska Island Marine	Summer	**0.519**	**0.771**		0.003	-0.037	
Aleutian Islands	LEAU	Segula & Davidof Islands Colonies	Summer	**0.410**	**0.597**		0.059	-0.048	
Aleutian Islands	LESP	Buldir Island Colony	Summer	**0.583**	**1.171**		**-0.411**	-0.022	
Aleutian Islands	NOFU	Chagulak Island Marine	Summer	**3.215**	**1.580**			0.028	
Aleutian Islands	NOFU	Chagulak Island Marine	Winter	**3.217**	**1.421**			**-2.549**	
Aleutian Islands	NOFU	Seguam Island Marine	Summer	**2.880**	**1.224**			0.151	
Aleutian Islands	PAAU	Buldir & Near Islands Marine	Summer	**0.681**	**1.237**		**-0.293**	-0.009	
Aleutian Islands	PAAU	Buldir Island Colony	Summer	**0.583**	**1.171**		**-0.411**	-0.022	
Aleutian Islands	PAAU	Fenimore Pass & Atka Island Marine	Summer	**1.922**	**0.945**		**-0.156**	**-0.102**	
Aleutian Islands	PAAU	Gareloi Island Marine	Summer	**1.097**	**0.924**		0.167	-0.082	
Aleutian Islands	RLKI	Buldir Island Colony	Summer	**0.583**	**1.171**			-0.022	
Aleutian Islands	WHAU	Amchitka Pass 180W51N	Summer	**0.725**	**0.647**		0.232	**-0.159**	

LME	Species	IBA	Season	Shallow SWT	Deep SWT	Sea Ice Cover	Large Copepods	Euphausiids	Benthic Infauna
Aleutian Islands	WHAU	Buldir & Near Islands Marine	Summer	**0.601**	**1.149**		**-0.246**	0.032	
Aleutian Islands	WHAU	Buldir Island Colony	Summer	**0.583**	**1.171**		**-0.411**	-0.022	
Aleutian Islands	WHAU	Fenimore Pass & Atka Island Marine	Summer	**2.129**	**0.839**		0.241	0.151	
Aleutian Islands	WHAU	Fenimore Pass & Atka Island Marine	Summer	**2.019**	**0.938**		-0.044	-0.080	
Aleutian Islands	WHAU	Gareloi Island Marine	Summer	**1.076**	**0.925**		0.216	-0.027	
Aleutian Islands	WHAU	Kagamil Island Marine	Summer	**2.891**	**1.991**		0.221	0.165	
Aleutian Islands	WHAU	Kiska Island Marine	Summer	**0.396**	**0.562**		0.078	0.013	
Aleutian Islands	WHAU	Seguam Island Marine	Summer	**2.945**	**1.246**		0.252	0.122	
Eastern Bering Sea	BLKI	Cape Peirce & Cape Newenham	Summer	**0.681**		**-0.890**		0.350	
Eastern Bering Sea	BLKI	Ilnik Marine	Summer	**0.380**				0.187	
Eastern Bering Sea	BLKI	Izembek Lagoon & Bechevin Bay	Summer	**0.540**				**-0.139**	
Eastern Bering Sea	BLKI	St. Matthew & Hall Islands Colonies	Summer	**0.549**				0.021	
Eastern Bering Sea	BLKI	Unimak & Akutan Passes	Summer	**0.835**	**0.544**			-0.075	
Eastern Bering Sea	BLSC	Northern Alaska Peninsula Coastal	Spring	**0.363**					-0.013
Eastern Bering Sea	BLSC	Northern Alaska Peninsula Coastal	Spring	**0.405**					0.088
Eastern Bering Sea	BLSC	Port Moller	Fall	**0.398**					-0.064
Eastern Bering Sea	BLSC	Port Moller	Spring	**0.398**					-0.007
Eastern Bering Sea	CRAU	St. Matthew & Hall Islands Colonies	Summer	**0.549**			0.503	0.021	
Eastern Bering Sea	CRAU	Unimak & Akutan Passes	Winter	**0.834**	**0.576**		-0.034	**-0.584**	
Eastern Bering Sea	FTSP	Bering Sea Shelf Edge 173W58N	Summer	**0.561**	**0.425**		-0.041	0.184	
Eastern Bering Sea	FTSP	Bering Sea Shelf Edge 178W61N	Summer	**0.391**	**0.193**	**-0.581**	0.185	0.367	
Eastern Bering Sea	GLGU	Bering Sea Shelf 170W58N	Winter	**0.534**		**-0.988**		-0.068	
Eastern Bering Sea	GLGU	Bering Sea Shelf Edge 174W59N	Winter	**0.562**	**0.411**			**-0.933**	
Eastern Bering Sea	GWGU	Bering Sea Shelf 163W56N	Winter	**0.521**	**0.650**			**-0.284**	
Eastern Bering Sea	GWGU	Bering Sea Shelf 165W56N	Winter	**0.502**				**-0.377**	
Eastern Bering Sea	GWGU	Bering Sea Shelf 166W56N	Winter	**0.525**	**0.626**			**-0.315**	
Eastern Bering Sea	GWGU	Bering Sea Shelf 166W57N	Winter	**0.524**	**0.601**			**-0.139**	
Eastern Bering Sea	GWGU	Bering Sea Shelf Edge 166W55N	Winter	**0.671**	**0.534**			**-0.463**	
Eastern Bering Sea	GWGU	Bering Sea Shelf Edge 168W56N	Winter	**0.544**	**0.571**			**-0.120**	

LME	Species	IBA	Season	Shallow SWT	Deep SWT	Sea Ice Cover	Large Copepods	Euphausiids	Benthic Infauna
Eastern Bering Sea	GWGU	Cape Tanak Marine	Winter	**2.091**	**1.197**			**-0.991**	
Eastern Bering Sea	GWGU	Izembek Lagoon & Bechevin Bay	Fall	**0.546**				**-0.480**	
Eastern Bering Sea	GWGU	Northern Alaska Peninsula Coastal	Fall	**0.369**				-0.058	
Eastern Bering Sea	GWGU	Port Moller	Fall	**0.400**				**-0.124**	
Eastern Bering Sea	GWGU	Unimak & Akutan Passes	Summer	**0.837**	**0.544**			-0.075	
Eastern Bering Sea	KIEI	Cape Vancouver Marine	Spring	**0.906**		**-0.840**			0.037
Eastern Bering Sea	KIEI	Northern Alaska Peninsula Coastal	Spring	**0.407**					0.084
Eastern Bering Sea	KIEI	Nushagak & Kvichak Bays	Spring	**0.366**		**-0.813**			**-0.169**
Eastern Bering Sea	KIMU	Unimak & Akutan Passes	Summer	**1.503**	**0.938**			0.036	
Eastern Bering Sea	LEAU	St. Matthew & Hall Islands Colonies	Summer	**0.538**		**-0.770**	0.561	0.033	
Eastern Bering Sea	NOFU	Bering Sea Shelf Edge 174W59N	Summer	**0.591**	**0.416**			**-0.224**	
Eastern Bering Sea	NOFU	St. Matthew & Hall Islands Colonies	Summer	**0.549**				0.021	
Eastern Bering Sea	PAAU	St. Matthew & Hall Islands Colonies	Summer	**0.538**		**-0.770**	0.561	0.033	
Eastern Bering Sea	PAAU	St. Paul Island Colony	Summer	**0.502**			-0.001	0.162	
Eastern Bering Sea	RLKI	St. George Island Marine	Summer	**0.552**	**0.497**			0.237	
Eastern Bering Sea	RLKI	St. George Island Marine	Winter	**0.528**	**0.514**			**-0.441**	
Eastern Bering Sea	SOSH	Unimak & Akutan Passes	Summer	**0.777**	**0.538**			-0.035	
Eastern Bering Sea	STEI	Izembek Lagoon & Bechevin Bay	Fall	**0.554**					**-0.178**
Eastern Bering Sea	STEI	Izembek Lagoon & Bechevin Bay	Spring	**0.556**					**-0.163**
Eastern Bering Sea	STEI	Jacksmith Bay to Cape Pierce	Spring	**0.683**		**-0.808**			-0.075
Eastern Bering Sea	STEI	Kuskokwim Bay	Spring	**0.915**		**-0.722**			-0.081
Eastern Bering Sea	STEI	Northern Alaska Peninsula Coastal	Fall	**0.366**					0.050
Eastern Bering Sea	STEI	Northern Alaska Peninsula Coastal	Spring	**0.363**					0.016
Eastern Bering Sea	STEI	Port Moller	Fall	**0.381**					-0.099
Eastern Bering Sea	STEI	Port Moller	Spring	**0.400**					-0.008
Eastern Bering Sea	STSH	Unimak & Akutan Passes	Summer	**0.805**	**0.535**			-0.053	
Eastern Bering Sea	TBMU	St. Matthew & Hall Islands Colonies	Summer	**0.549**				0.021	
Eastern Bering Sea	WHAU	Unimak & Akutan Passes	Summer	**0.983**	**0.612**		0.015	0.015	
Eastern Bering Sea	WHAU	Unimak & Akutan Passes	Winter	**1.106**	**0.675**		**-0.104**	**-0.455**	

LME	Species	IBA	Season	Shallow SWT	Deep SWT	Sea Ice Cover	Large Copepods	Euphausiids	Benthic Infauna
Eastern Bering Sea	WWSC	Ilnik Marine	Winter	**0.391**					-0.011
Eastern Bering Sea	WWSC	Northern Alaska Peninsula Coastal	Spring	**0.359**					0.001
Northern Bering Sea	BLKI	Diomede Islands Colonies	Summer	**0.245**				0.177	
Northern Bering Sea	CRAU	Diomede Islands Colonies	Summer	**0.200**		**-0.218**	0.087	0.196	
Northern Bering Sea	CRAU	Savoonga Colonies	Summer	**0.350**		0.044	0.330	0.333	
Northern Bering Sea	CRAU	Southwest Cape Colonies	Summer	**0.474**		**-0.435**	0.581	0.679	
Northern Bering Sea	CRAU	Western St. Lawrence Island Marine	Summer	**0.480**		**-0.232**	0.575	0.607	
Northern Bering Sea	LEAU	Savoonga Colonies	Summer	**0.346**		0.007	0.285	0.323	
Northern Bering Sea	PAAU	Bering Strait	Summer	**0.161**		**-0.198**	0.099	0.241	
Northern Bering Sea	PAAU	Diomede Islands Colonies	Summer	**0.200**		**-0.218**	0.087	0.196	
Northern Bering Sea	PAAU	King Island Colony	Summer	**0.478**			0.099	0.252	
Northern Bering Sea	SPEI	East Norton Sound	Summer	**0.273**		**-0.699**			0.234
Northern Bering Sea	SPEI	St. Lawrence Island Polynya	Winter	**0.735**		**-0.769**			-0.016
Northern Bering Sea	SPEI	Western St. Lawrence Island Marine	Summer	**0.455**		**-0.596**			0.120
Gulf of Alaska	ALTE	Eastern Kodiak Island Marine	Summer	**0.315**				0.049	
Gulf of Alaska	ANMU	Castle Rock Colonies	Summer	**0.418**				0.009	
Gulf of Alaska	ANMU	Cherni Island Complex Colonies	Summer	**0.537**				0.032	
Gulf of Alaska	ANMU	Shumagin Islands Marine	Summer	**0.490**	**0.515**			0.009	
Gulf of Alaska	BLKI	Barren Islands Colonies	Summer	**0.291**				0.099	
Gulf of Alaska	BLKI	Castle Rock Colonies	Summer	**0.422**				0.008	
Gulf of Alaska	BLKI	Semidi Islands Colonies	Summer	**0.431**	**0.453**			0.018	
Gulf of Alaska	BLKI	Spitz Island Colony	Summer	**0.399**				0.059	
Gulf of Alaska	BLSC	Kachemak Bay	Winter	**0.250**					-0.022
Gulf of Alaska	BLSC	Marmot Bay	Winter	**0.349**					**-0.180**
Gulf of Alaska	CAAU	Castle Rock Colonies	Summer	**0.418**			0.004	0.009	
Gulf of Alaska	CAAU	Cherni Island Complex Colonies	Summer	**0.541**			0.074	0.028	
Gulf of Alaska	CAAU	Gulf of Alaska Shelf 155W57N	Summer	**0.362**	**0.399**		0.070	0.095	
Gulf of Alaska	GWGU	Bird Island Colony	Summer	**0.607**				0.049	
Gulf of Alaska	GWGU	Cape Douglas to Amalik Bay	Fall	**0.329**	**0.270**			0.087	

**LME**	**Species**	**IBA**	**Season**	**Shallow SWT**	**Deep SWT**	**Sea Ice Cover**	**Large Copepods**	**Euphausiids**	**Benthic Infauna**
Gulf of Alaska	GWGU	Cold & Morzhovoi Bays	Summer	**0.521**				0.017	
Gulf of Alaska	GWGU	Gulf of Alaska Shelf 151W58N	Winter	**0.258**	**0.317**			-0.027	
Gulf of Alaska	GWGU	Gulf of Alaska Shelf Edge 163W54N	Winter	**0.779**	**0.565**			**-0.341**	
Gulf of Alaska	GWGU	Kenai Fjords	Fall	**0.313**	**0.202**			**-0.102**	
Gulf of Alaska	GWGU	Kenai Fjords	Summer	**0.313**				0.137	
Gulf of Alaska	GWGU	Lower Cook Inlet 153W59N	Winter	**0.281**				**-0.476**	
Gulf of Alaska	GWGU	Sanak Islands Marine	Summer	**0.671**	**0.539**			0.010	
Gulf of Alaska	HADU	Cape Douglas to Amalik Bay	Fall	**0.338**	**0.285**				-0.033
Gulf of Alaska	KIMU	Kachemak Bay	Summer	**0.259**				0.119	
Gulf of Alaska	LESP	Cherni Island Complex Colonies	Summer	**0.533**			0.072	0.051	
Gulf of Alaska	MAMU	Kachemak Bay	Summer	**0.243**				0.113	
Gulf of Alaska	NOFU	Semidi Islands Colonies	Summer	**0.451**				0.010	
Gulf of Alaska	PAAU	Castle Rock Colonies	Summer	**0.418**			0.004	0.009	
Gulf of Alaska	PAAU	Koniuji-Shumagin Islands Colonies	Summer	**0.534**	**0.557**		0.032	0.029	
Gulf of Alaska	PAAU	Semidi Islands Colonies	Summer	**0.451**			0.006	0.010	
Gulf of Alaska	PAAU	Shumagin Islands Marine	Summer	**0.534**	**0.557**		0.032	0.029	
Gulf of Alaska	PIGU	Kenai Fjords	Summer	**0.317**	**0.240**				0.109
Gulf of Alaska	STEI	Chiniak Bay	Winter	**0.313**					**-0.108**
Gulf of Alaska	STEI	Clam Gulch	Spring	**0.266**					**-0.103**
Gulf of Alaska	STEI	Kamishak Bay	Spring	**0.288**					**-0.140**
Gulf of Alaska	STEI	Sitkinak Strait	Spring	**0.357**					**-0.221**
Gulf of Alaska	WWSC	Cape Douglas to Amalik Bay	Winter	**0.333**					**-0.133**
Gulf of Alaska	WWSC	Eastern Kodiak Island Marine	Winter	**0.279**	**0.367**				0.016
Gulf of Alaska	WWSC	Kachemak Bay	Winter	**0.265**					-0.065
Number climate vulnerable				**138/138 (100%)**	**68/68 (100%)**	**17/19 (89%)**	**9/46 (20%)**	**22/107 (21%)**	**9/31 (29%)**
