# Supplementary material for: An assessment of climate change vulnerability for Important Bird Areas in the Bering Sea and Aleutian Arc
Source: PLoS One. 2019 Apr 17;14(4):e0214573. doi: 10.1371/journal.pone.0214573 (PMC6469780; doi:10.1371/journal.pone.0214573)
Supplement: S4 Table — (PDF) [file pone.0214573.s004.pdf]

S4 Table. Magnitude-agreement scores, by species core area. Values in bold indicate core areas that exceeded the climate vulnerability threshold.

LME	Species	IBA	Season	Shallow SWT	Deep SWT	Sea Ice Cover	Large Copepods	Euphausiids	Benthic Infauna
Aleutian Islands	ANMU	Buldir & Near Islands Marine	Summer	**0.670**	**1.061**			0.000	
Aleutian Islands	ANMU	Buldir Island Colony	Summer	**0.389**	**0.781**			-0.007	
Aleutian Islands	ANMU	Fenimore Pass & Atka Island Marine	Summer	**2.237**	**0.879**			0.089	
Aleutian Islands	ANMU	Koniuji-Atka Island Colony	Summer	**2.054**	**0.867**			0.069	
Aleutian Islands	BLKI	Buldir Island Colony	Summer	**0.389**	**0.781**			-0.007	
Aleutian Islands	CRAU	Buldir Island Colony	Summer	**0.389**	**0.781**		**-0.274**	-0.007	
Aleutian Islands	CRAU	Gareloi Island Marine	Summer	**1.074**	**0.887**		0.066	-0.002	
Aleutian Islands	FTSP	Buldir Island Colony	Summer	**0.389**	**0.781**		**-0.274**	-0.007	
Aleutian Islands	FTSP	Koniuji-Atka Island Colony	Summer	**2.054**	**0.867**		0.053	0.069	
Aleutian Islands	GWGU	Buldir & Near Islands Marine	Summer	0.055	**0.416**			0.008	
Aleutian Islands	GWGU	Fenimore Pass & Atka Island Marine	Summer	**2.239**	**0.892**			0.091	
Aleutian Islands	GWGU	Fenimore Pass & Atka Island Marine	Summer	**1.935**	**0.926**			-0.054	
Aleutian Islands	GWGU	Fenimore Pass & Atka Island Marine	Winter	**2.142**	**0.929**			**-0.561**	
Aleutian Islands	GWGU	Kiska Island Colonies	Summer	**0.272**	**0.389**			0.018	
Aleutian Islands	LEAU	Gareloi Island Marine	Summer	**1.066**	**0.883**		0.066	-0.002	
Aleutian Islands	LEAU	Kiska Island Marine	Summer	**0.346**	**0.514**		0.001	-0.012	
Aleutian Islands	LEAU	Segula & Davidof Islands Colonies	Summer	**0.137**	**0.398**		0.020	-0.016	
Aleutian Islands	LESP	Buldir Island Colony	Summer	**0.389**	**0.781**		**-0.274**	-0.007	
Aleutian Islands	NOFU	Chagulak Island Marine	Summer	**3.215**	**1.580**			0.000	
Aleutian Islands	NOFU	Chagulak Island Marine	Winter	**3.217**	**1.421**			**-2.549**	
Aleutian Islands	NOFU	Seguam Island Marine	Summer	**2.880**	**1.224**			0.050	
Aleutian Islands	PAAU	Buldir & Near Islands Marine	Summer	**0.454**	**0.825**		-0.098	-0.006	
Aleutian Islands	PAAU	Buldir Island Colony	Summer	**0.389**	**0.781**		**-0.274**	-0.007	
Aleutian Islands	PAAU	Fenimore Pass & Atka Island Marine	Summer	**1.922**	**0.945**		-0.052	-0.068	
Aleutian Islands	PAAU	Gareloi Island Marine	Summer	**1.097**	**0.924**		0.056	-0.027	
Aleutian Islands	RLKI	Buldir Island Colony	Summer	**0.389**	**0.781**			-0.007	
Aleutian Islands	WHAU	Amchitka Pass 180W51N	Summer	**0.483**	**0.432**		0.077	-0.053	
Aleutian Islands	WHAU	Buldir & Near Islands Marine	Summer	**0.401**	**0.766**		-0.082	0.011	

LME	Species	IBA	Season	Shallow SWT	Deep SWT	Sea Ice Cover	Large Copepods	Euphausiids	Benthic Infauna
Aleutian Islands	WHAU	Buldir Island Colony	Summer	**0.389**	**0.781**		**-0.274**	-0.007	
Aleutian Islands	WHAU	Fenimore Pass & Atka Island Marine	Summer	**2.129**	**0.839**		0.080	0.101	
Aleutian Islands	WHAU	Fenimore Pass & Atka Island Marine	Summer	**2.019**	**0.938**		-0.015	-0.053	
Aleutian Islands	WHAU	Gareloi Island Marine	Summer	**1.076**	**0.925**		0.072	-0.009	
Aleutian Islands	WHAU	Kagamil Island Marine	Summer	**2.891**	**1.991**		0.000	0.000	
Aleutian Islands	WHAU	Kiska Island Marine	Summer	**0.264**	**0.375**		0.026	0.004	
Aleutian Islands	WHAU	Seguam Island Marine	Summer	**2.945**	**1.246**		0.084	0.041	
Eastern Bering Sea	BLKI	Cape Peirce & Cape Newenham	Summer	**0.681**		**-0.890**		0.000	
Eastern Bering Sea	BLKI	Ilnik Marine	Summer	**0.380**				0.000	
Eastern Bering Sea	BLKI	Izembek Lagoon & Bechevin Bay	Summer	**0.540**				-0.093	
Eastern Bering Sea	BLKI	St. Matthew & Hall Islands Colonies	Summer	**0.549**				0.014	
Eastern Bering Sea	BLKI	Unimak & Akutan Passes	Summer	**0.835**	**0.544**			-0.025	
Eastern Bering Sea	BLSC	Northern Alaska Peninsula Coastal	Spring	**0.242**					-0.004
Eastern Bering Sea	BLSC	Northern Alaska Peninsula Coastal	Spring	**0.405**					0.029
Eastern Bering Sea	BLSC	Port Moller	Fall	**0.398**					-0.021
Eastern Bering Sea	BLSC	Port Moller	Spring	**0.398**					-0.002
Eastern Bering Sea	CRAU	St. Matthew & Hall Islands Colonies	Summer	**0.549**			0.000	0.014	
Eastern Bering Sea	CRAU	Unimak & Akutan Passes	Winter	**0.834**	**0.576**		-0.011	**-0.584**	
Eastern Bering Sea	FTSP	Bering Sea Shelf Edge 173W58N	Summer	**0.561**	**0.283**		-0.014	0.123	
Eastern Bering Sea	FTSP	Bering Sea Shelf Edge 178W61N	Summer	**0.391**	**0.129**	**-0.581**	0.062	0.000	
Eastern Bering Sea	GLGU	Bering Sea Shelf 170W58N	Winter	**0.534**		**-0.988**		-0.045	
Eastern Bering Sea	GLGU	Bering Sea Shelf Edge 174W59N	Winter	**0.562**	**0.274**			**-0.933**	
Eastern Bering Sea	GWGU	Bering Sea Shelf 163W56N	Winter	**0.521**	**0.650**			-0.095	
Eastern Bering Sea	GWGU	Bering Sea Shelf 165W56N	Winter	**0.502**				**-0.126**	
Eastern Bering Sea	GWGU	Bering Sea Shelf 166W56N	Winter	**0.525**	**0.626**			**-0.105**	
Eastern Bering Sea	GWGU	Bering Sea Shelf 166W57N	Winter	**0.524**	**0.601**			-0.046	
Eastern Bering Sea	GWGU	Bering Sea Shelf Edge 166W55N	Winter	**0.671**	**0.534**			**-0.463**	
Eastern Bering Sea	GWGU	Bering Sea Shelf Edge 168W56N	Winter	**0.544**	**0.571**			-0.040	
Eastern Bering Sea	GWGU	Cape Tanak Marine	Winter	**2.091**	**1.197**			**-0.991**	

LME	Species	IBA	Season	Shallow SWT	Deep SWT	Sea Ice Cover	Large Copepods	Euphausiids	Benthic Infauna
Eastern Bering Sea	GWGU	Izembek Lagoon & Bechevin Bay	Fall	**0.546**				**-0.320**	
Eastern Bering Sea	GWGU	Northern Alaska Peninsula Coastal	Fall	**0.369**				-0.019	
Eastern Bering Sea	GWGU	Port Moller	Fall	**0.400**				-0.041	
Eastern Bering Sea	GWGU	Unimak & Akutan Passes	Summer	**0.837**	**0.544**			-0.025	
Eastern Bering Sea	KIEI	Cape Vancouver Marine	Spring	**0.906**		**-0.840**			0.025
Eastern Bering Sea	KIEI	Northern Alaska Peninsula Coastal	Spring	**0.407**					0.028
Eastern Bering Sea	KIEI	Nushagak & Kvichak Bays	Spring	**0.366**		**-0.813**			**-0.112**
Eastern Bering Sea	KIMU	Unimak & Akutan Passes	Summer	**1.503**	**0.938**			0.012	
Eastern Bering Sea	LEAU	St. Matthew & Hall Islands Colonies	Summer	**0.538**		**-0.770**	0.000	0.011	
Eastern Bering Sea	NOFU	Bering Sea Shelf Edge 174W59N	Summer	**0.591**	**0.277**			**-0.149**	
Eastern Bering Sea	NOFU	St. Matthew & Hall Islands Colonies	Summer	**0.549**				0.014	
Eastern Bering Sea	PAAU	St. Matthew & Hall Islands Colonies	Summer	**0.538**		**-0.770**	0.000	0.011	
Eastern Bering Sea	PAAU	St. Paul Island Colony	Summer	**0.502**			0.000	0.054	
Eastern Bering Sea	RLKI	St. George Island Marine	Summer	**0.552**	**0.497**			0.079	
Eastern Bering Sea	RLKI	St. George Island Marine	Winter	**0.528**	**0.514**			**-0.147**	
Eastern Bering Sea	SOSH	Unimak & Akutan Passes	Summer	**0.777**	**0.538**			-0.012	
Eastern Bering Sea	STEI	Izembek Lagoon & Bechevin Bay	Fall	**0.554**					**-0.119**
Eastern Bering Sea	STEI	Izembek Lagoon & Bechevin Bay	Spring	**0.556**					-0.054
Eastern Bering Sea	STEI	Jacksmith Bay to Cape Pierce	Spring	**0.683**		**-0.808**			-0.025
Eastern Bering Sea	STEI	Kuskokwim Bay	Spring	**0.915**		**-0.722**			-0.054
Eastern Bering Sea	STEI	Northern Alaska Peninsula Coastal	Fall	**0.366**					0.017
Eastern Bering Sea	STEI	Northern Alaska Peninsula Coastal	Spring	**0.363**					0.005
Eastern Bering Sea	STEI	Port Moller	Fall	**0.381**					-0.033
Eastern Bering Sea	STEI	Port Moller	Spring	**0.400**					-0.003
Eastern Bering Sea	STSH	Unimak & Akutan Passes	Summer	**0.805**	**0.535**			-0.018	
Eastern Bering Sea	TBMU	St. Matthew & Hall Islands Colonies	Summer	**0.549**				0.014	
Eastern Bering Sea	WHAU	Unimak & Akutan Passes	Summer	**0.983**	**0.612**		0.005	0.000	
Eastern Bering Sea	WHAU	Unimak & Akutan Passes	Winter	**1.106**	**0.675**		-0.035	**-0.455**	
Eastern Bering Sea	WWSC	Ilnik Marine	Winter	**0.391**					-0.004

LME	Species	IBA	Season	Shallow SWT	Deep SWT	Sea Ice Cover	Large Copepods	Euphausiids	Benthic Infauna
Eastern Bering Sea	WWSC	Northern Alaska Peninsula Coastal	Spring	**0.239**					0.000
Northern Bering Sea	BLKI	Diomede Islands Colonies	Summer	**0.245**				0.000	
Northern Bering Sea	CRAU	Diomede Islands Colonies	Summer	**0.133**		**-0.145**	0.029	0.000	
Northern Bering Sea	CRAU	Savoonga Colonies	Summer	**0.350**		0.015	0.110	0.000	
Northern Bering Sea	CRAU	Southwest Cape Colonies	Summer	**0.316**		**-0.435**	0.000	0.000	
Northern Bering Sea	CRAU	Western St. Lawrence Island Marine	Summer	**0.320**		**-0.154**	0.000	0.202	
Northern Bering Sea	LEAU	Savoonga Colonies	Summer	**0.346**		0.002	0.095	0.000	
Northern Bering Sea	PAAU	Bering Strait	Summer	**0.107**		**-0.132**	0.033	0.000	
Northern Bering Sea	PAAU	Diomede Islands Colonies	Summer	**0.133**		**-0.145**	0.029	0.000	
Northern Bering Sea	PAAU	King Island Colony	Summer	**0.478**			0.000	0.000	
Northern Bering Sea	SPEI	East Norton Sound	Summer	0.091		**-0.466**			0.000
Northern Bering Sea	SPEI	St. Lawrence Island Polynya	Winter	**0.735**		**-0.769**			-0.005
Northern Bering Sea	SPEI	Western St. Lawrence Island Marine	Summer	**0.303**		**-0.596**			0.000
Gulf of Alaska	ALTE	Eastern Kodiak Island Marine	Summer	**0.315**				0.016	
Gulf of Alaska	ANMU	Castle Rock Colonies	Summer	**0.279**				0.003	
Gulf of Alaska	ANMU	Cherni Island Complex Colonies	Summer	**0.537**				0.011	
Gulf of Alaska	ANMU	Shumagin Islands Marine	Summer	**0.490**	**0.343**			0.000	
Gulf of Alaska	BLKI	Barren Islands Colonies	Summer	**0.194**				0.000	
Gulf of Alaska	BLKI	Castle Rock Colonies	Summer	**0.282**				0.003	
Gulf of Alaska	BLKI	Semidi Islands Colonies	Summer	**0.287**	**0.302**			0.000	
Gulf of Alaska	BLKI	Spitz Island Colony	Summer	**0.266**				0.000	
Gulf of Alaska	BLSC	Kachemak Bay	Winter	**0.166**					-0.007
Gulf of Alaska	BLSC	Marmot Bay	Winter	**0.349**					**-0.120**
Gulf of Alaska	CAAU	Castle Rock Colonies	Summer	**0.279**			0.000	0.003	
Gulf of Alaska	CAAU	Cherni Island Complex Colonies	Summer	**0.541**			0.025	0.009	
Gulf of Alaska	CAAU	Gulf of Alaska Shelf 155W57N	Summer	**0.241**	**0.399**		0.000	0.000	
Gulf of Alaska	GWGU	Bird Island Colony	Summer	**0.607**				0.016	
Gulf of Alaska	GWGU	Cape Douglas to Amalik Bay	Fall	**0.219**	**0.270**			0.000	
Gulf of Alaska	GWGU	Cold & Morzhovoi Bays	Summer	**0.521**				0.006	

LME	Species	IBA	Season	Shallow SWT	Deep SWT	Sea Ice Cover	Large Copepods	Euphausiids	Benthic Infauna
Gulf of Alaska	GWGU	Gulf of Alaska Shelf 151W58N	Winter	**0.172**	**0.212**			-0.018	
Gulf of Alaska	GWGU	Gulf of Alaska Shelf Edge 163W54N	Winter	**0.779**	**0.565**			**-0.227**	
Gulf of Alaska	GWGU	Kenai Fjords	Fall	**0.209**	0.067			-0.034	
Gulf of Alaska	GWGU	Kenai Fjords	Summer	**0.209**				0.000	
Gulf of Alaska	GWGU	Lower Cook Inlet 153W59N	Winter	**0.187**				**-0.317**	
Gulf of Alaska	GWGU	Sanak Islands Marine	Summer	**0.671**	**0.539**			0.000	
Gulf of Alaska	HADU	Cape Douglas to Amalik Bay	Fall	**0.225**	**0.285**				-0.011
Gulf of Alaska	KIMU	Kachemak Bay	Summer	**0.172**				0.000	
Gulf of Alaska	LESP	Cherni Island Complex Colonies	Summer	**0.533**			0.024	0.017	
Gulf of Alaska	MAMU	Kachemak Bay	Summer	**0.162**				0.000	
Gulf of Alaska	NOFU	Semidi Islands Colonies	Summer	**0.301**				0.000	
Gulf of Alaska	PAAU	Castle Rock Colonies	Summer	**0.279**			0.000	0.003	
Gulf of Alaska	PAAU	Koniuji-Shumagin Islands Colonies	Summer	**0.534**	**0.557**		0.000	0.000	
Gulf of Alaska	PAAU	Semidi Islands Colonies	Summer	**0.301**			0.000	0.000	
Gulf of Alaska	PAAU	Shumagin Islands Marine	Summer	**0.534**	**0.557**		0.000	0.000	
Gulf of Alaska	PIGU	Kenai Fjords	Summer	**0.212**	0.080				0.036
Gulf of Alaska	STEI	Chiniak Bay	Winter	**0.313**					-0.036
Gulf of Alaska	STEI	Clam Gulch	Spring	**0.177**					-0.069
Gulf of Alaska	STEI	Kamishak Bay	Spring	**0.192**					-0.093
Gulf of Alaska	STEI	Sitkinak Strait	Spring	**0.238**					**-0.148**
Gulf of Alaska	WWSC	Cape Douglas to Amalik Bay	Winter	**0.222**					-0.089
Gulf of Alaska	WWSC	Eastern Kodiak Island Marine	Winter	**0.186**	**0.367**				0.005
Gulf of Alaska	WWSC	Kachemak Bay	Winter	**0.177**					-0.022
Number climate vulnerable				**136/138 (99%)**	**66/68 (97%)**	**17/19 (89%)**	**5/46 (11%)**	**14/107 (13%)**	**4/31 (13%)**
