# Supplementary material for: An assessment of climate change vulnerability for Important Bird Areas in the Bering Sea and Aleutian Arc
Source: PLoS One. 2019 Apr 17;14(4):e0214573. doi: 10.1371/journal.pone.0214573 (PMC6469780; doi:10.1371/journal.pone.0214573)

S1 Figs. Climate vulnerability maps by variable and model

Shallow Sea Water Temperature (SWT)

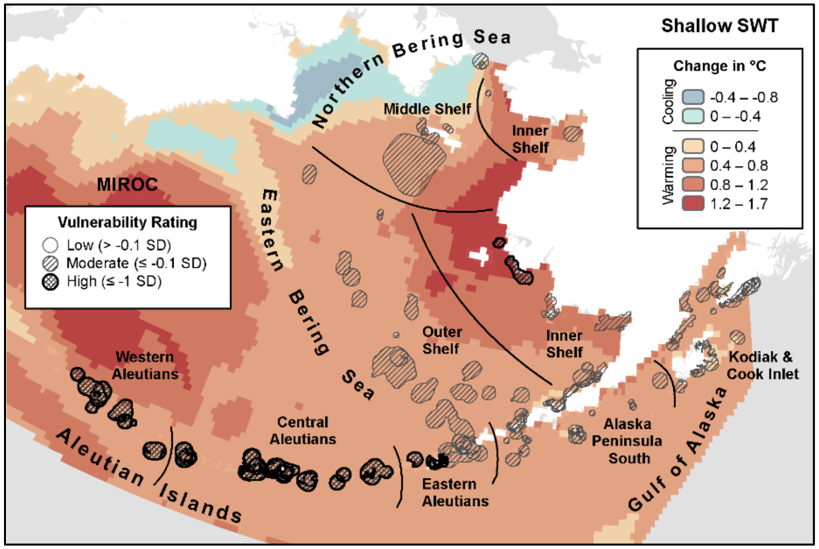

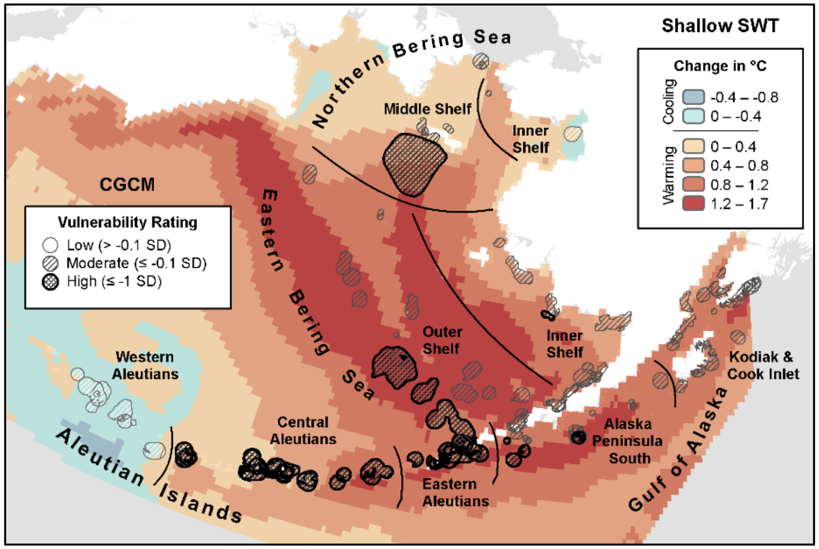

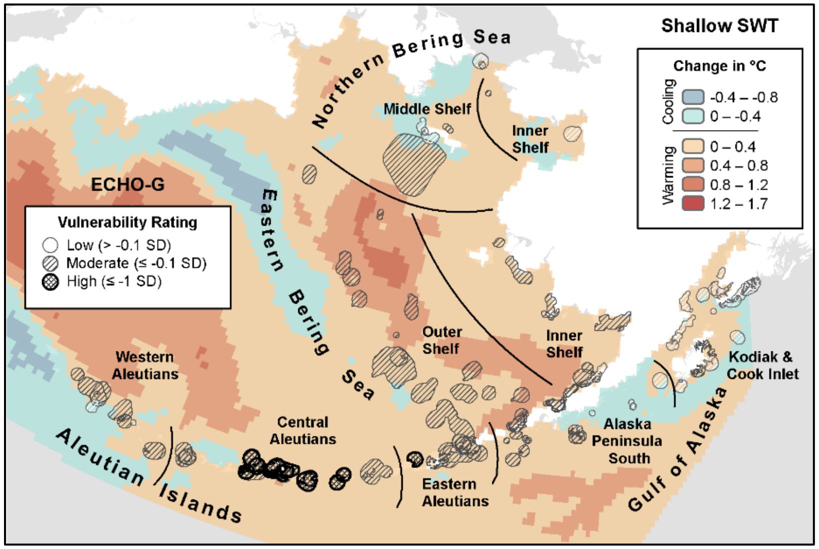

S1 Figs. Climate vulnerability maps by variable and model

Deep Sea Water Temperature (SWT)

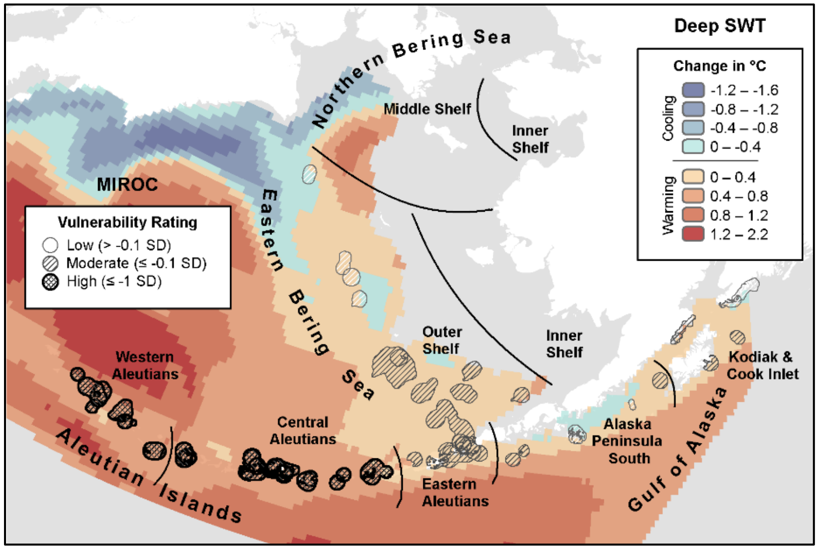

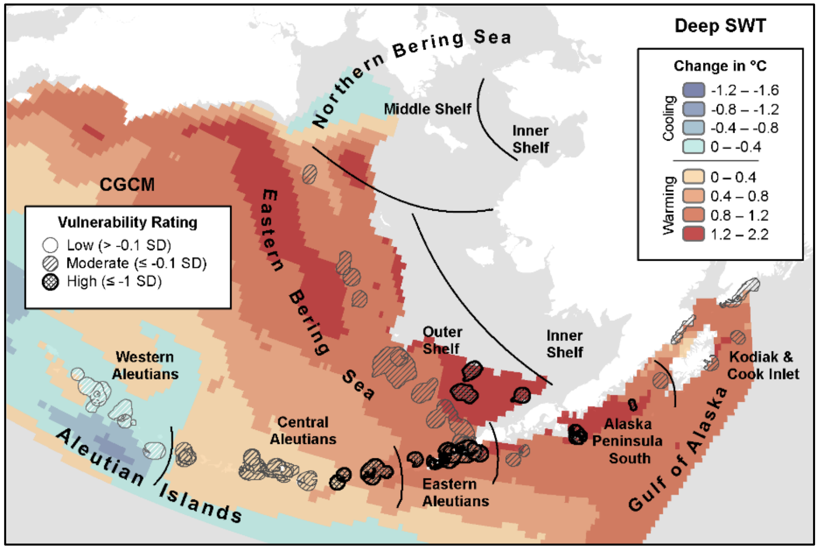

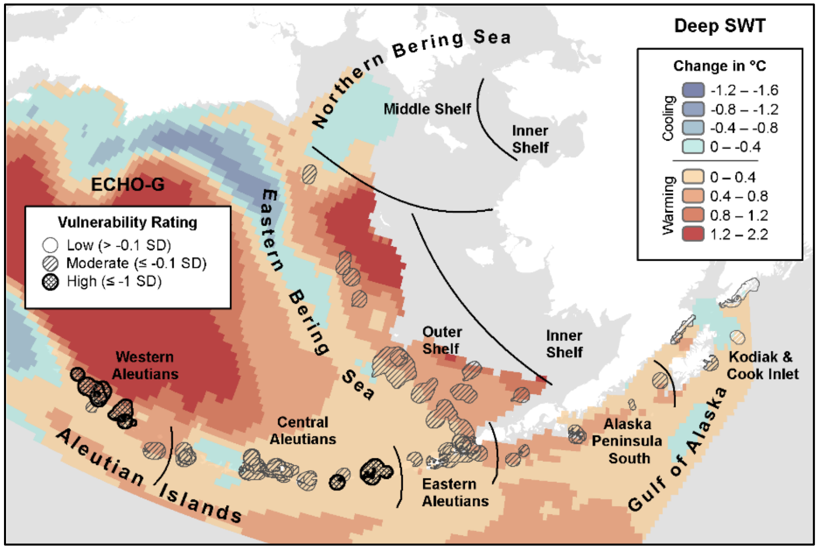

S1 Figs. Climate vulnerability maps by variable and model

Sea Ice

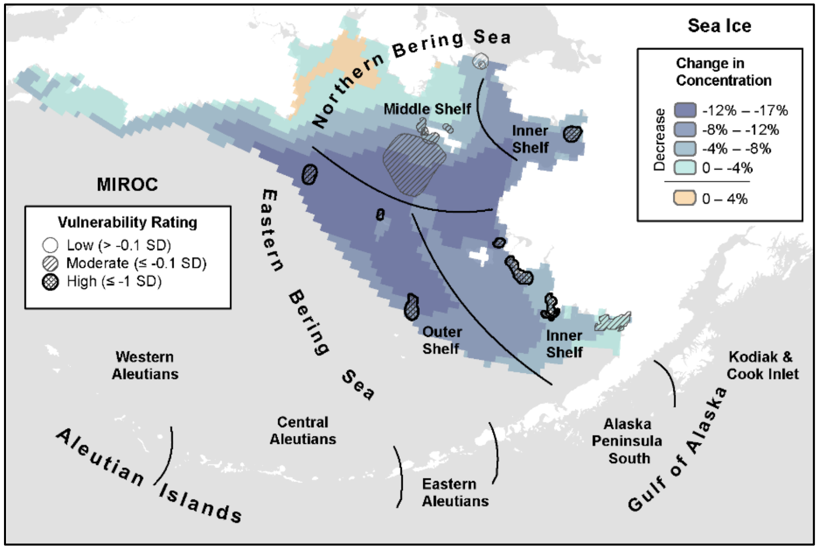

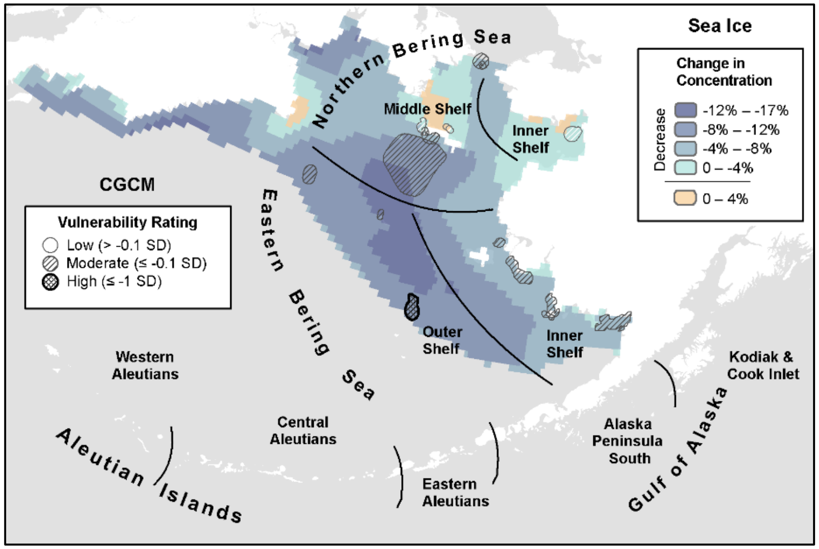

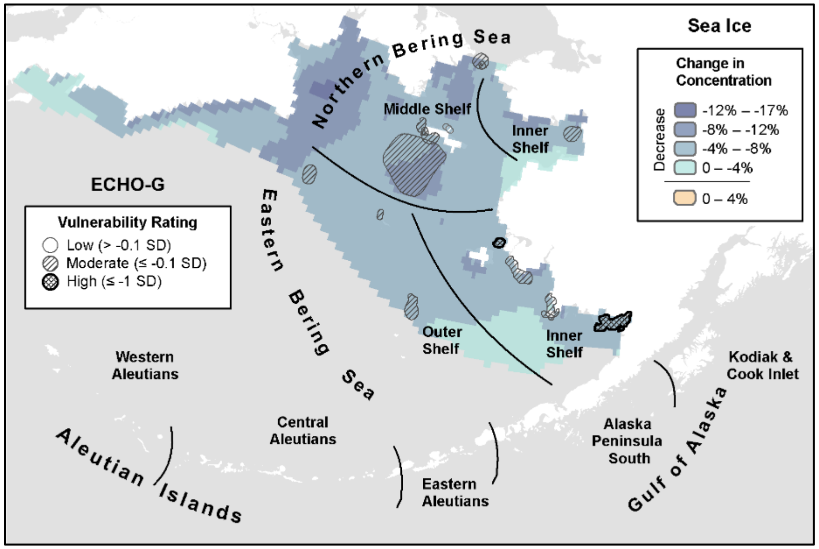

S1 Figs. Climate vulnerability maps by variable and model

Large Copepods

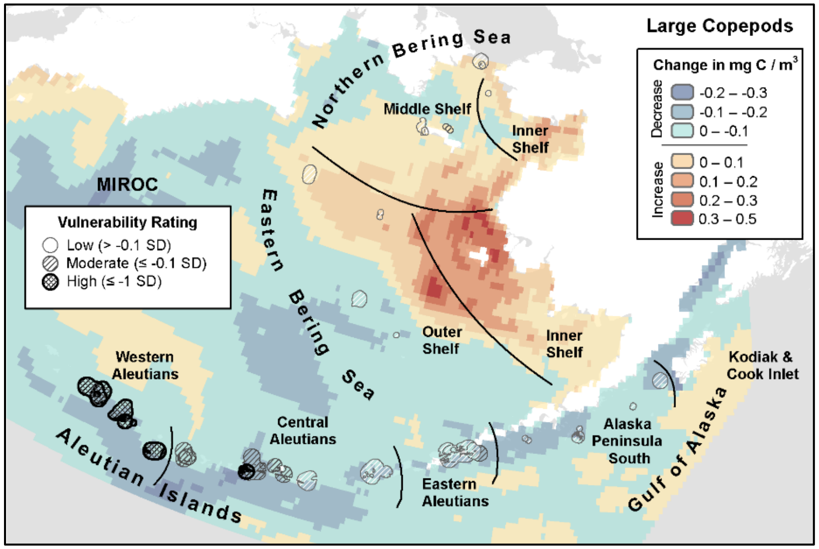

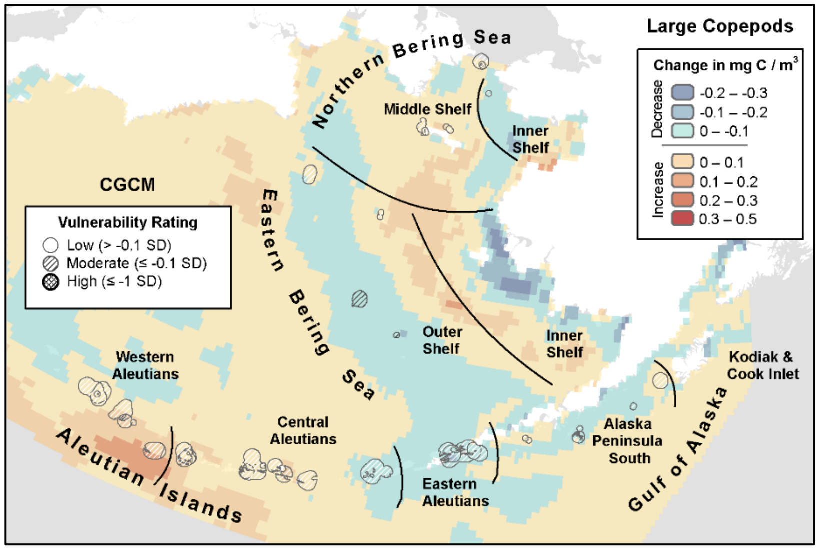

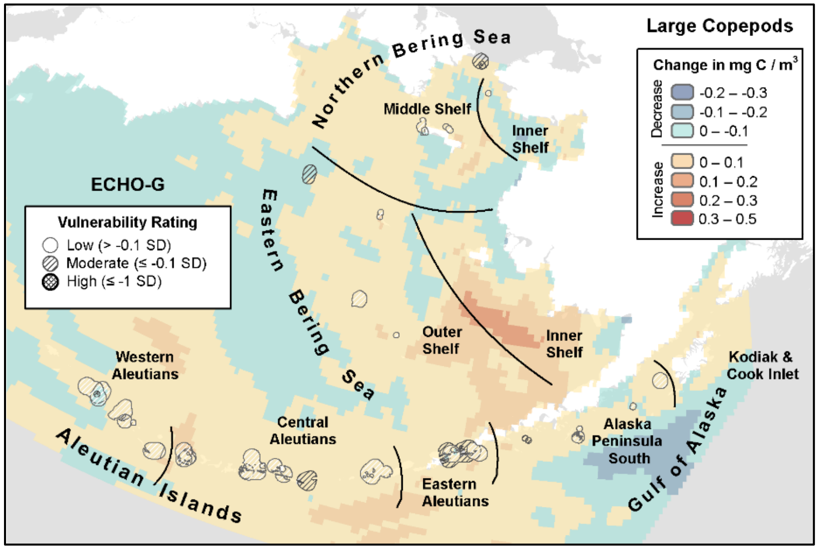

S1 Figs. Climate vulnerability maps by variable and model

Euphausiids

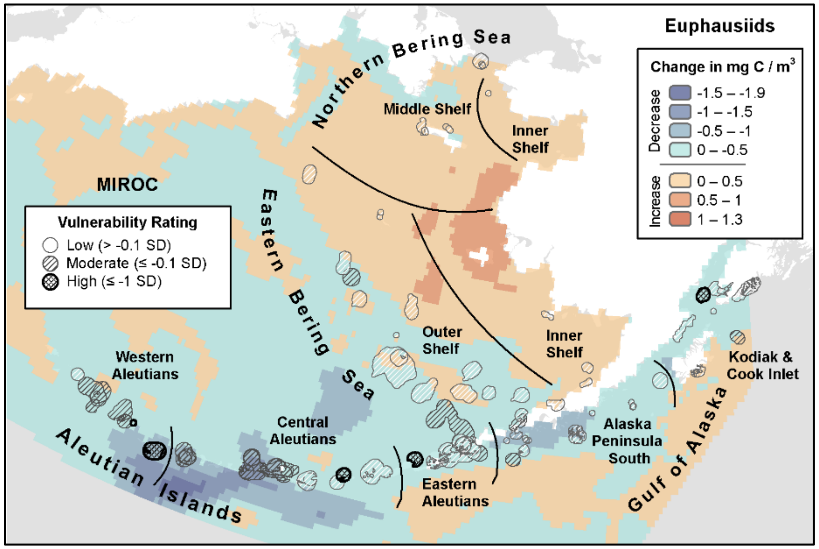

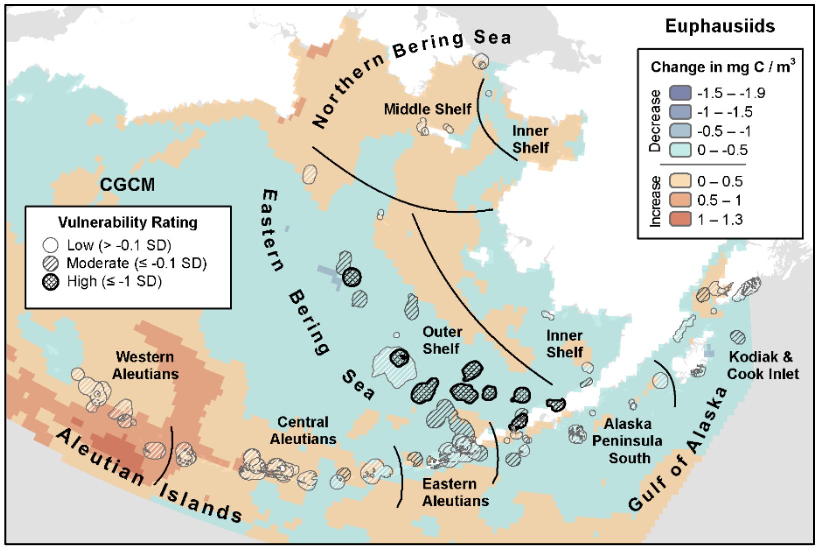

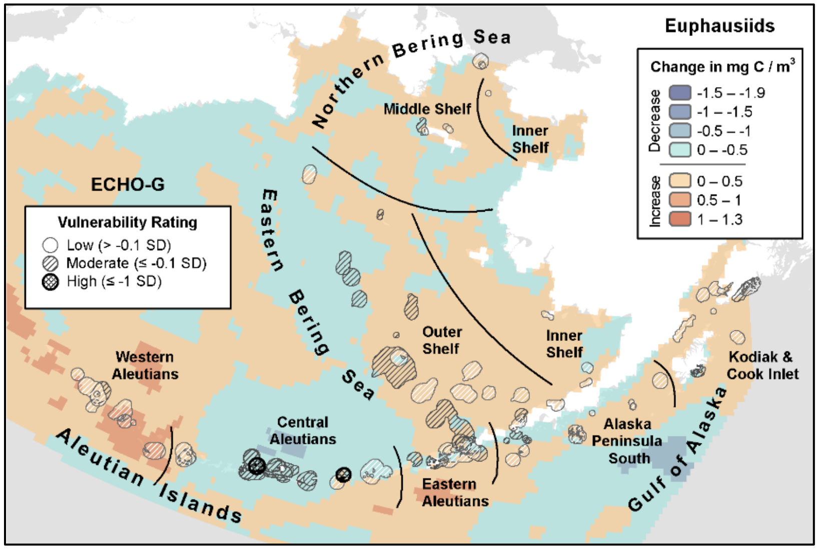

S1 Figs. Climate vulnerability maps by variable and model

Benthic Infauna

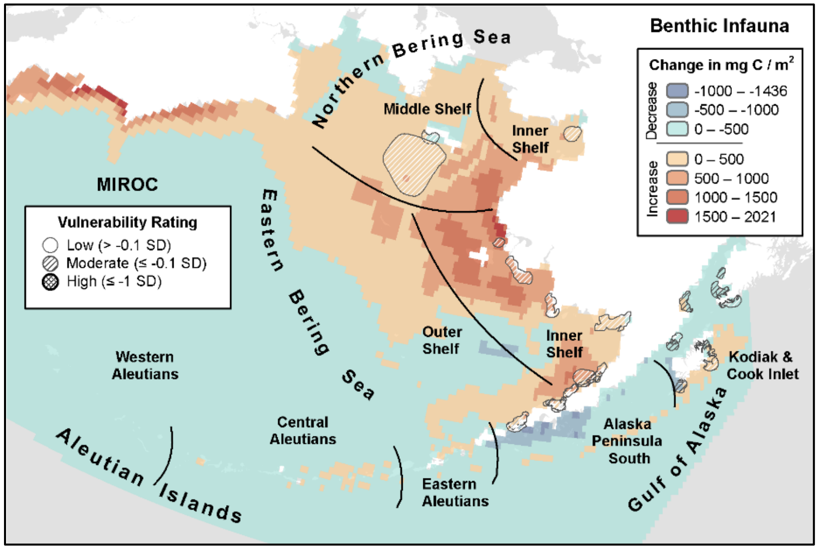

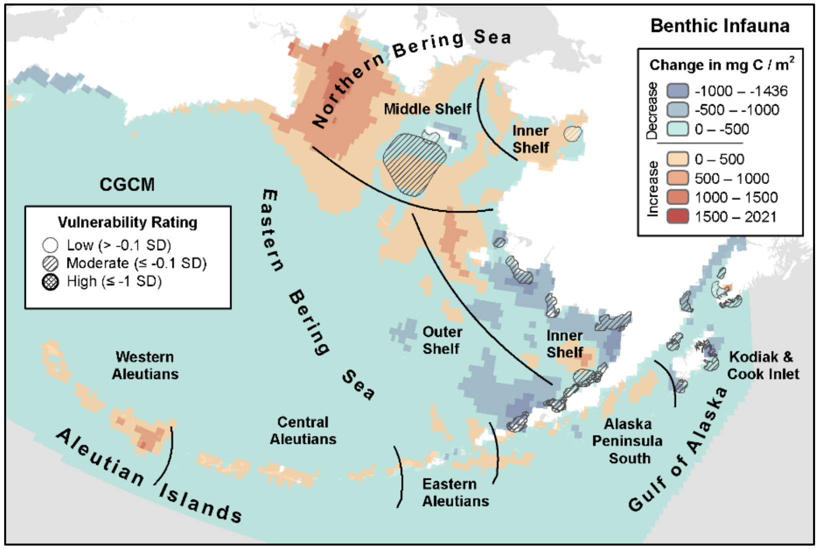

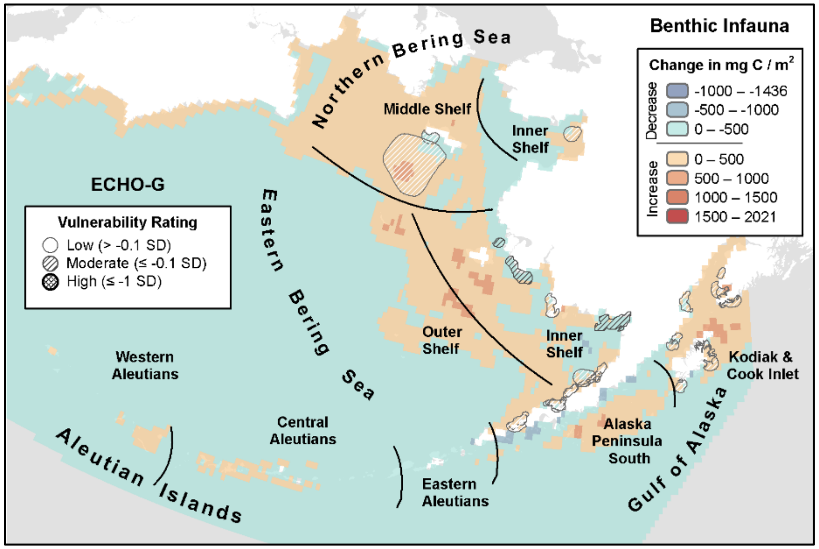

Supplement: S1 Figs — (PDF) [file pone.0214573.s005.pdf]
